# Supplementary material for: The relationship between parenting engagement and academic performance
Source: Sci Rep. 2022 Dec 24;12:22300. doi: 10.1038/s41598-022-26258-z (PMC9789521; doi:10.1038/s41598-022-26258-z)
Supplement: Supplementary file 1 — Supplementary Information. [file 41598_2022_26258_MOESM1_ESM.docx]

Supplementary Materials

Detailed Methods

**Comparison of respondents, sample and population.** In total, 17,519 researchers responded, of which 14,910 were considered eligible in that they finished survey as well as reported having children. Further data processing was performed to exclude incomplete and anonymous responses, reducing the sample size to 11,226, or 75.3% of eligible responses. The highest missing rates occurred in questions asking about the birth year of children (n=2,358), followed by the year when respondents’ partner taking parental leaves (n=566) and the year when respondents taking parental leaves (n=387). 59 respondents were also removed as they were not associated with an email address to which we had sent the survey. As one of the key variables analyzed in this paper is gender, respondents who did not provide gender information (men or women), n=16 respondents identified as *other* gender, as well as those who lacked disciplinary information were excluded.

On the whole, the final sample analyzed contained 10,445 researchers, which represents 70.1% of all researchers who completed the survey. This accounts for 0.70% of the set of sampled researchers, and for 0.40% of the entire population. Information found on researchers’ publication records allows us to compare the characteristics of respondents with those of the sample and the population. These are not the self-declared country, discipline, and gender but, rather, those that were obtained from researchers’ publication records. Table S6 compares the number and percentage of respondents, by main country of affiliation, defined as the country to which authors were affiliated most often in their publications (with random assignation in case of draws). It shows that English-speaking countries have had a much higher response rate than the average (United States [1.64%], United Kingdom [1.19%], Canada [1.48%], Australia [1.23%], New Zealand [2.06%]). These five countries account for more than 65% of all responses.

Most European countries exhibit a lower-than-average response rate—such as Germany [0.45%], France [0.44%], Spain [0.46%] and Italy [0.45%]—which may be due to a more negative perception of unsolicited surveys, as exemplified by the General Data Protection Regulation adopted in 2018. Norway [1.29%] is the only European country with a response rate above 1%, with Sweden [0.97%] and Finland [0.96%] not being far from 1%. Given the access limitations of the Qualtrics tool in China, a very low response rate [0.02%] was obtained for researchers from that country, and a similar percentage was obtained for Japanese researchers [0.04%]. Response rates for India [0.08%], Brazil [0.14%], Russia [0.16%] and Turkey [0.18%] are also low. Therefore, the survey has a strong North American and European focus, and is much less representative of Asia and the Global South.

We also observe disparities in terms of response rates by disciplines (Table S7), defined as the main discipline in which the researcher is active. Sampled researchers associated with disciplines of the social sciences and humanities, such as Psychology [1.97%], Social Sciences [1.61%], Professional Fields [1.53%], Health [1.29%] and Humanities [1.07%] have a higher-than-average response rate. Response rate was slightly below average for researchers in the medical sciences (Biomedical Research [0.65%], Clinical Medicine [0.64%]) and much below average for the natural sciences, with Engineering [0.26%], Chemistry [0.32%], and Physics [0.34%] being two times lower than the average. This may also be a function of the different percentage of women in those disciplines: women are more likely to be active in disciplines of the social sciences and much less in the natural sciences. This may also be a function of the topic of the survey: as shown in Table S8, the response rate of women was almost three times higher than that of men [1.34% vs. 0.53%].

Globally, the comparison of bibliometric-retrieved sociodemographic variables match with those that were declared by respondents. Participants declared to be overwhelmingly from the United States (n=5,197), with the UK (n=972), Canada (n=642) and Australia (n=421) also being well-represented. Within Europe, Germany was most highly represented (n=345), followed by France (n=233), The Netherlands (n=218), Italy (n=204) and Spain (n=195). Our respondents consisted of 57.7% women, and 42.3% men. Of these, 93.1% were in partnered relationships at the moment, of which 98.5% were in heterosexual partnership, and had, on average, two children. Each participant had an average of 34.2 publications (SD=59.9) of which seven were first authored (SD=19.4), and 3.4 were last-authored (SD=5.8) (Table S9). The average year of their first publication was 2003 with 2017 being the year of their last publication.

**Parenting types.** We overlaid self-identification of parenting engagement with marital status to construct categorizations for parenting types (Table S10). The parenting type classification system assumes that for every Lead parent, there must be at least one Satellite parent; and that a Satellite parent must indicate the presence of a Lead parent. In contrast, the presence of a dual parent, must also indicate the presence of at least one other dual parent. For the sake of the analysis, the sole parent category was combined with other data under the Lead parent category as it was assumed that sole parents were, like lead parents, primarily responsible for the majority of childcare.

**Indicators of productivity and esteem.** Based on researcher’s publication profiles found on the WoS for the 1980-2017, several bibliometric indicators were compiled. Research productivity was defined as the number of papers to which they contributed over their career. Articles were also analyzed as a function of author position: first, middle and last. Annual numbers of papers were also compiled. Impact was defined through two citation measures: the total number of citations (TCS) received by papers to which they contributed, as well as the Mean Normalized Citation Score (MNCS) (39), which is a field- and year-normalized citation indicator that takes into account the fact that the expected number of citations for a paper (and therefore a researcher) varies as a function of discipline and publication year.

**Quantitative analysis.** The analytic set for multiple linear regression models includes 10,013 respondents after excluding 432 with zero MNCS. The outcome variables are the number of publications indexed in WOS between 1980 and 2017 in the productivity model. For the impact model, both the number of total citations (TCS) and mean normalized citation scores (MNCS) are used as proxies for research impact. Natural log transformation was applied to normalize the indicators. The main variables of interest are parenting engagement and the length of parental leaves. We identified three parenting types based on respondents’ caregiving situation (either current or when their children were dependents). Lead parents are the primary caregivers to their children, dual parents share equal parenting roles with either their partner or non-parental caregivers (e.g., grandparents, nannies), and satellite parents whose partner or non-parental others are primary caregivers for children. The lengths of parental leaves are divided into six categories: no leave, less than one month, at least one but less than three months, three to six months, more than six but less than 12 months, and 12 months or more.

To test whether the relationship between parental engagement and research performance differs by gender and partnership status, we include a three-way interaction term between parenting type, partnership status, and gender. Partnership status is determined by the current occupation of respondents’ partner to be in an academic or non-academic sector. Academic partner is strictly referred to respondents whose partner is employed in academic sectors for research and/or teaching. Partners employed in government, private, or other sectors, as well as retired and not employed, are all designated as non-academic partner (see Table S11–S12 for the composition of employment sectors between respondents and their partner). To make our analysis more inclusive, single parents are assumed to be lead parents and therefore were retained in the sample.

Considering the strong presence of the US respondents in our sample and the fact that US is the only advanced country without a nation-wide parental leave policy, we split the sample into the US-only and non-US subsets when modeling the effect of parental leave lengths on productivity (see Table S13–S14 for the distribution of parenting type and parental leave length by gender, among US-only and non-US samples). In addition to the above-mentioned three-way interaction between parenting type, partnership status, and gender, we also include an interaction term between gender and parental leave lengths to examine whether the relationship between parental leave lengths and productivity differs by gender.

Informed by the prior literature, we control for the number of children as well as respondents’ academic age, highest degree earned, employment sector, and primary discipline. Academic age is calculated based on the first and last years of publication plus one. Given that the effect of age is often multiplicative, we include academic age in its polynomial form and center the variable at the mean to render the intercept more meaningful and interpretable. Both the highest degree and employment sector are binary variables, indicating whether respondents hold a doctoral degree and work in an academic sector for research and/or teaching. Number of children is a nominal variable with four possible values: 1, 2, 3, as well as 4 and more; so is the main discipline, which is regrouped from the original 14 disciplines into four: Arts and Humanities, Health Sciences, Natural Sciences, and Social Sciences.

The general form of the model is written as:

log(Y)=β_0_+βX+ϵ.

The intercept (β_0_) represents women as a dual parent with a non-academic partner and two children, whose publication career is 13.4 years long and who hold a doctoral degree and an academic employment in social sciences.

Since we are interested in the gendered division of parenting labor among academic couples who assume similar responsibilities at work, we also fit the models with a subset of respondents in the two-academics household. Noted that both partnership status and respondent’s employment are removed in the models which includes only academic respondents with an academic partner.

**Qualitative analysis.** A free text section was included at the end of the survey that encouraged participants to “Please feel free to add any additional comments you have regarding childcare and scientific labor, drawing upon your own experiences”. In total, 5,976 participants completed this section. To analyze this large number of responses, analysis was separated into a 2-stage coding approach.

Stage 1 coding involved a random sample of 1000 comments beinginductively coded using NVivo (Figure S5) into themes using a grounded theory-informed approach using a line-by-line analysis approach to identify the main themes *(46–48)*. All inductive codes were then collapsed into 59 overarching axial codes, and further to 8 Stage 2 codes (Table S18) which allows for the continued manual coding a large number of remaining responses (n=4976 comments).

Stage 2 coding was conducted in Excel to allow for swift and robust coding of the remaining responses. An additional ‘Other’ category was permitted to retain data variability and richness as well as to elicit further codes as they gained prominence, in addition extensive memo-making and reflexive note-taking during Stage 2 (Figure S6).. The choice to continue Stage 2 coding manually was to ensure that an appropriate level of sensitivity and nuance to allow the data to speak and give voice to members of the research community.

We drew exemplar quotes from the dominant themes to complement quantitative analyses at the end of stage 2. Selection of these quotes was performed to ensure fair representation of men and women’s voices, to ensure global coverage, as well as those that were considered by the research team as representative of the sentiment represented by the Stage 2 code definition. However, the quotes here should be read as illustrative, but not a comprehensive reporting of the qualitative component of the survey.

Additional Analysis

**Comparison of the modeling results with the full sample (n=10,445) versus the reduced sample (n=10,013).** Among the effects that are statistically significant with the reduced sample at the 0.05 level, the positive effects of being a man (β=0.05, *p*=0.025) and being a satellite parent (β=0.08, *p*=0.004) remain significant with the full sample, yet the negative effects of being a single parent (β=−0.14, *p*=0.089) and a lead parent with an academic partner (β=−0.10, *p*=0.055) are only significant with the full sample at the 0.1 level (Table S15, Column 1–2). Furthermore, the significant positive effect of having an academic partner with the reduced sample at the 0.1 level (β=0.05, *p*=0.082) is no longer significant with the full sample (β=0.04, *p*=0.116). Conversely, the negative effect of being a lead father turns significant at the 0.1 level (β=−0.13, *p*=0.086).

The modeling results on TCS with full and reduced samples are largely the same, including the positive effect of having an academic partner for women (β=0.12, *p*=0.012) and the additional negative effect of being a lead father (β=−0.40, *p*=−0.009) (Table S15, Column 3–4). The only exception is that the additional negative effect of being a lead mother with an academic partner is significant only at the 0.1 level with the full sample (β=−0.17, *p*=0.066), as compared to the 0.05 level with the reduced sample (β=−0.22, *p*=0.022).

Overall, it seems that utilizing the full sample (including 432 respondents having zero MNCS) alleviates the negative effects, possibly due to the spread of the number of papers these 432 respondents have published ranging from 1 to 35, though 83% of them having less than four papers and half of them only one single publication.

**Comparison of the TCS model with the inclusion of the productivity measure versus without.** The impact of the number of papers on the total citations is not large but statistically significant (β=0.01, *p*<0.001) (Table S15, Column 5). The biggest difference between the two models is the effect of academic employment, which is statistically significant in the model whose predictors excluding the productivity measure (β=−0.11, *p*<0.001), but virtually nonexistent once the number of papers is included in the model (β=−0.01, *p*=0.812). This suggests that much of these two effects overlaps quite a bit, so the effect of academic employment can be largely explained by one’s productivity. Another noticeable difference is that the strongly negative effect of being a single mom and satellite parent at the same time is statistically significant in the model including the productivity measure (β=−0.58, *p*=0.044), but not in the model without (β=−0.52, *p*=0.143).

**Leave Models.** Analysis by parental and partner types allows for comparison with the previous results. We find that the positive effects of being men (β=0.15, *p*=0.001) and a satellite parent (β=0.10, *p*=0.02) are only significant in the US-only sample, suggesting that the significance of these two effects in the full model is primary driven by the US respondents (Table S5). The same applies to the positive effect of having an academic partner (β=0.07, *p*=0.081) at the 0.01 significance level. In addition, the previously significant negative effects of being a single parent as well as the interaction between lead parent and academic partner is gone in both US-only and non-US samples. It is worth noting, however, that being a single, satellite mother among non-US respondents suffers an additional 39.9% decrease in productivity (β=−0.51, *p*=0.048) whereas a single, satellite father gains an additional 64.6% increase (β=0.50, *p*=0.099), though the latter is not statistically significant.


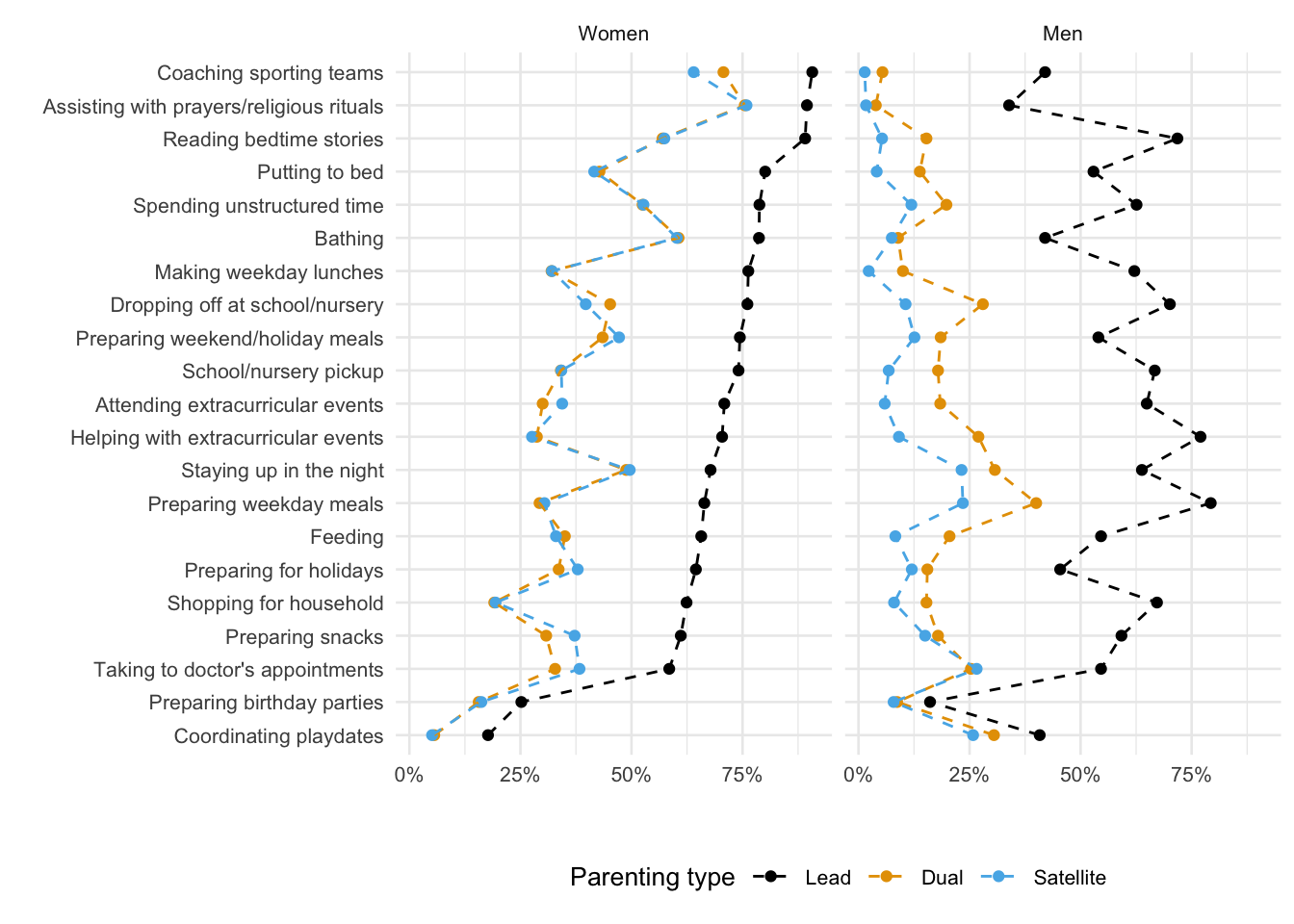


Fig. S1. Percentage of respondents being the primary caregivers in parenting activities, by gender and parenting type.

Table S1. Frequencies and proportions of being primary caregivers in parenting-related activities by gender and parenting type (n=10,445).

| Activity | Primary | Lead | | | Dual | | | Satellite | | |
| --- | --- | --- | --- | --- | --- | --- | --- | --- | --- | --- |
|  |  | W | M | *p* | W | M | *p* | W | M | *p* |
| Coordinating playdates for child(ren) | No | 171 (9.3) | 101 (58.0) | <0.001 | 918 (29.3) | 2390 (94.6) | <0.001 | 378 (36.0) | 1693 (98.6) | <0.001 |
|  | Yes | 1676 (90.7) | 73 (42.0) |  | 2214 (70.7) | 136 (5.4) |  | 671 (64.0) | 24 (1.4) |  |
| Preparing birthday parties and other child events | No | 194 (10.5) | 115 (66.1) | <0.001 | 767 (24.5) | 2428 (96.1) | <0.001 | 253 (24.1) | 1687 (98.3) | <0.001 |
|  | Yes | 1653 (89.5) | 59 (33.9) |  | 2365 (75.5) | 98 (3.9) |  | 796 (75.9) | 30 (1.7) |  |
| Attending extracurricular events | No | 537 (29.1) | 61 (35.1) | *0.122* | 2193 (70.0) | 2062 (81.6) | <0.001 | 688 (65.6) | 1615 (94.1) | <0.001 |
|  | Yes | 1310 (70.9) | 113 (64.9) |  | 939 (30.0) | 464 (18.4) |  | 361 (34.4) | 102 (5.9) |  |
| Helping child(ren) with extracurricular events | No | 479 (25.9) | 58 (33.3) | *0.05* | 2064 (65.9) | 2074 (82.1) | <0.001 | 690 (65.8) | 1600 (93.2) | <0.001 |
|  | Yes | 1368 (74.1) | 116 (66.7) |  | 1068 (34.1) | 452 (17.9) |  | 359 (34.2) | 117 (6.8) |  |
| Coaching a child's sporting team | No | 1520 (82.3) | 103 (59.2) | ***<0.001*** | 2958 (94.4) | 1756 (69.5) | ***<0.001*** | 995 (94.9) | 1274 (74.2) | ***<0.001*** |
|  | Yes | 327 (17.7) | 71 (40.8) |  | 174 (5.6) | 770 (30.5) |  | 54 (5.1) | 443 (25.8) |  |
| Assisting with prayers/religious rituals | No | 1382 (74.8) | 146 (83.9) | 0.014 | 2642 (84.4) | 2305 (91.3) | <0.001 | 879 (83.8) | 1581 (92.1) | <0.001 |
|  | Yes | 465 (25.2) | 28 (16.1) |  | 490 (15.6) | 221 (8.7) |  | 170 (16.2) | 136 (7.9) |  |
| Preparing for holidays | No | 394 (21.3) | 101 (58.0) | <0.001 | 1233 (39.4) | 2301 (91.1) | <0.001 | 417 (39.8) | 1588 (92.5) | <0.001 |
|  | Yes | 1453 (78.7) | 73 (42.0) |  | 1899 (60.6) | 225 (8.9) |  | 632 (60.2) | 129 (7.5) |  |
| Spending unstructured time with your child(ren) | No | 694 (37.6) | 57 (32.8) | *0.258* | 2533 (80.9) | 2140 (84.7) | <0.001 | 845 (80.6) | 1580 (92.0) | <0.001 |
|  | Yes | 1153 (62.4) | 117 (67.2) |  | 599 (19.1) | 386 (15.3) |  | 204 (19.4) | 137 (8.0) |  |
| Dropping of child(ren) at school/nursery | No | 621 (33.6) | 36 (20.7) | ***0.001*** | 2213 (70.7) | 1515 (60.0) | ***<0.001*** | 730 (69.6) | 1313 (76.5) | <0.001 |
|  | Yes | 1226 (66.4) | 138 (79.3) |  | 919 (29.3) | 1011 (40.0) |  | 319 (30.4) | 404 (23.5) |  |
| Picking up child(ren) from school/nursery | No | 547 (29.6) | 40 (23.0) | *0.101* | 2232 (71.3) | 1843 (73.0) | *0.168* | 760 (72.4) | 1561 (90.9) | <0.001 |
|  | Yes | 1300 (70.4) | 134 (77.0) |  | 900 (28.7) | 683 (27.0) |  | 289 (27.6) | 156 (9.1) |  |
| Shopping for household/child(ren) | No | 391 (21.2) | 65 (37.4) | <0.001 | 1487 (47.5) | 2027 (80.2) | <0.001 | 496 (47.3) | 1513 (88.1) | <0.001 |
|  | Yes | 1456 (78.8) | 109 (62.6) |  | 1645 (52.5) | 499 (19.8) |  | 553 (52.7) | 204 (11.9) |  |
| Taking child(ren) to doctor's appointments | No | 202 (10.9) | 49 (28.2) | <0.001 | 1346 (43.0) | 2140 (84.7) | <0.001 | 447 (42.6) | 1626 (94.7) | <0.001 |
|  | Yes | 1645 (89.1) | 125 (71.8) |  | 1786 (57.0) | 386 (15.3) |  | 602 (57.4) | 91 (5.3) |  |
| Cooking and preparing weekday meals | No | 441 (23.9) | 52 (29.9) | *0.11* | 1715 (54.8) | 1818 (72.0) | <0.001 | 633 (60.3) | 1535 (89.4) | <0.001 |
|  | Yes | 1406 (76.1) | 122 (70.1) |  | 1417 (45.2) | 708 (28.0) |  | 416 (39.7) | 182 (10.6) |  |
| Cooking and preparing weekend/holiday meals | No | 595 (32.2) | 63 (36.2) | *0.35* | 1603 (51.2) | 1750 (69.3) | <0.001 | 529 (50.4) | 1319 (76.8) | <0.001 |
|  | Yes | 1252 (67.8) | 111 (63.8) |  | 1529 (48.8) | 776 (30.7) |  | 520 (49.6) | 398 (23.2) |  |
| Making weekday lunches | No | 634 (34.3) | 79 (45.4) | 0.008 | 2037 (65.0) | 2007 (79.5) | <0.001 | 703 (67.0) | 1574 (91.7) | <0.001 |
|  | Yes | 1213 (65.7) | 95 (54.6) |  | 1095 (35.0) | 519 (20.5) |  | 346 (33.0) | 143 (8.3) |  |
| Preparing snacks for child(ren) | No | 368 (19.9) | 82 (47.1) | <0.001 | 1793 (57.2) | 2178 (86.2) | <0.001 | 613 (58.4) | 1647 (95.9) | <0.001 |
|  | Yes | 1479 (80.1) | 92 (52.9) |  | 1339 (42.8) | 348 (13.8) |  | 436 (41.6) | 70 (4.1) |  |
| Feeding child(ren) | No | 438 (23.7) | 66 (37.9) | <0.001 | 2131 (68.0) | 2274 (90.0) | <0.001 | 712 (67.9) | 1677 (97.7) | <0.001 |
|  | Yes | 1409 (76.3) | 108 (62.1) |  | 1001 (32.0) | 252 (10.0) |  | 337 (32.1) | 40 (2.3) |  |
| Bathing child(ren) | No | 656 (35.5) | 95 (54.6) | <0.001 | 2079 (66.4) | 2135 (84.5) | <0.001 | 651 (62.1) | 1511 (88.0) | <0.001 |
|  | Yes | 1191 (64.5) | 79 (45.4) |  | 1053 (33.6) | 391 (15.5) |  | 398 (37.9) | 206 (12.0) |  |
| Reading bedtime stories | No | 767 (41.5) | 79 (45.4) | *0.354* | 2106 (67.2) | 1888 (74.7) | <0.001 | 647 (61.7) | 1260 (73.4) | <0.001 |
|  | Yes | 1080 (58.5) | 95 (54.6) |  | 1026 (32.8) | 638 (25.3) |  | 402 (38.3) | 457 (26.6) |  |
| Staying up in the night with child(ren) | No | 472 (25.6) | 80 (46.0) | <0.001 | 1770 (56.5) | 2058 (81.5) | <0.001 | 554 (52.8) | 1500 (87.4) | <0.001 |
|  | Yes | 1375 (74.4) | 94 (54.0) |  | 1362 (43.5) | 468 (18.5) |  | 495 (47.2) | 217 (12.6) |  |
| Putting child(ren) to bed | No | 718 (38.9) | 71 (40.8) | *0.622* | 2166 (69.2) | 2075 (82.1) | <0.001 | 659 (62.8) | 1459 (85.0) | <0.001 |
|  | Yes | 1129 (61.1) | 103 (59.2) |  | 966 (30.8) | 451 (17.9) |  | 390 (37.2) | 258 (15.0) |  |

Table S2. Frequencies and proportions of being primary caregivers in parenting-related activities among female academic respondents by academic partnership and parenting type (n=4,730).

| Activity | Primary | Lead | | | Dual | | | Satellite | | |
| --- | --- | --- | --- | --- | --- | --- | --- | --- | --- | --- |
|  |  | No | Yes | *p* | No | Yes | *p* | No | Yes | *p* |
| Coordinating playdates for child(ren) | No | 83 (9.1) | 30 (9.3) | 1 | 482 (29.0) | 281 (28.8) | 0.925 | 246 (38.4) | 66 (30.6) | 0.142 |
|  | Yes | 831 (90.9) | 292 (90.7) |  | 1179 (71.0) | 695 (71.2) |  | 395 (61.6) | 150 (69.4) |  |
| Preparing birthday parties and other child events | No | 100 (10.9) | 40 (12.4) | 0.831 | 394 (23.7) | 256 (26.2) | 0.211 | 167 (26.1) | 49 (22.7) | 0.588 |
|  | Yes | 814 (89.1) | 282 (87.6) |  | 1267 (76.3) | 720 (73.8) |  | 474 (73.9) | 167 (77.3) |  |
| Attending extracurricular events | No | 287 (31.4) | 113 (35.1) | 0.646 | 1155 (69.5) | 712 (73.0) | 0.123 | 428 (66.8) | 144 (66.7) | 1 |
|  | Yes | 627 (68.6) | 209 (64.9) |  | 506 (30.5) | 264 (27.0) |  | 213 (33.2) | 72 (33.3) |  |
| Helping child(ren) with extracurricular events | No | 263 (28.8) | 102 (31.7) | 0.714 | 1085 (65.3) | 681 (69.8) | **0.046** | 420 (65.5) | 143 (66.2) | 0.961 |
|  | Yes | 651 (71.2) | 220 (68.3) |  | 576 (34.7) | 295 (30.2) |  | 221 (34.5) | 73 (33.8) |  |
| Coaching a child's sporting team | No | 777 (85.0) | 274 (85.1) | 1 | 1568 (94.4) | 931 (95.4) | 0.319 | 606 (94.5) | 206 (95.4) | 0.907 |
|  | Yes | 137 (15.0) | 48 (14.9) |  | 93 (5.6) | 45 (4.6) |  | 35 (5.5) | 10 (4.6) |  |
| Assisting with prayers/religious rituals | No | 695 (76.0) | 260 (80.7) | 0.311 | 1387 (83.5) | 836 (85.7) | 0.207 | 544 (84.9) | 177 (81.9) | 0.588 |
|  | Yes | 219 (24.0) | 62 (19.3) |  | 274 (16.5) | 140 (14.3) |  | 97 (15.1) | 39 (18.1) |  |
| Preparing for holidays | No | 214 (23.4) | 86 (26.7) | 0.646 | 632 (38.0) | 417 (42.7) | **0.047** | 258 (40.2) | 87 (40.3) | 1 |
|  | Yes | 700 (76.6) | 236 (73.3) |  | 1029 (62.0) | 559 (57.3) |  | 383 (59.8) | 129 (59.7) |  |
| Spending unstructured time with your child(ren) | No | 390 (42.7) | 141 (43.8) | 0.923 | 1347 (81.1) | 808 (82.8) | 0.324 | 519 (81.0) | 172 (79.6) | 0.907 |
|  | Yes | 524 (57.3) | 181 (56.2) |  | 314 (18.9) | 168 (17.2) |  | 122 (19.0) | 44 (20.4) |  |
| Dropping off child(ren) at school/nursery | No | 312 (34.1) | 156 (48.4) | **0.001** | 1064 (64.1) | 789 (80.8) | **0.001** | 438 (68.3) | 170 (78.7) | 0.051 |
|  | Yes | 602 (65.9) | 166 (51.6) |  | 597 (35.9) | 187 (19.2) |  | 203 (31.7) | 46 (21.3) |  |
| Picking up child(ren) from school/nursery | No | 252 (27.6) | 135 (41.9) | **0.001** | 1114 (67.1) | 764 (78.3) | **0.001** | 457 (71.3) | 167 (77.3) | 0.26 |
|  | Yes | 662 (72.4) | 187 (58.1) |  | 547 (32.9) | 212 (21.7) |  | 184 (28.7) | 49 (22.7) |  |
| Shopping for household/child(ren) | No | 220 (24.1) | 86 (26.7) | 0.714 | 790 (47.6) | 479 (49.1) | 0.498 | 316 (49.3) | 96 (44.4) | 0.504 |
|  | Yes | 694 (75.9) | 236 (73.3) |  | 871 (52.4) | 497 (50.9) |  | 325 (50.7) | 120 (55.6) |  |
| Taking child(ren) to doctor's appointments | No | 99 (10.8) | 56 (17.4) | **0.011** | 670 (40.3) | 466 (47.7) | **0.002** | 290 (45.2) | 86 (39.8) | 0.418 |
|  | Yes | 815 (89.2) | 266 (82.6) |  | 991 (59.7) | 510 (52.3) |  | 351 (54.8) | 130 (60.2) |  |
| Cooking and preparing weekday meals | No | 229 (25.1) | 91 (28.3) | 0.646 | 891 (53.6) | 558 (57.2) | 0.135 | 386 (60.2) | 126 (58.3) | 0.907 |
|  | Yes | 685 (74.9) | 231 (71.7) |  | 770 (46.4) | 418 (42.8) |  | 255 (39.8) | 90 (41.7) |  |
| Cooking and preparing weekend/holiday meals | No | 337 (36.9) | 115 (35.7) | 0.923 | 823 (49.5) | 535 (54.8) | **0.035** | 319 (49.8) | 111 (51.4) | 0.907 |
|  | Yes | 577 (63.1) | 207 (64.3) |  | 838 (50.5) | 441 (45.2) |  | 322 (50.2) | 105 (48.6) |  |
| Making weekday lunches | No | 328 (35.9) | 119 (37.0) | 0.923 | 1072 (64.5) | 652 (66.8) | 0.315 | 452 (70.5) | 119 (55.1) | **0.004** |
|  | Yes | 586 (64.1) | 203 (63.0) |  | 589 (35.5) | 324 (33.2) |  | 189 (29.5) | 97 (44.9) |  |
| Preparing snacks for child(ren) | No | 194 (21.2) | 69 (21.4) | 1 | 937 (56.4) | 591 (60.6) | 0.083 | 383 (59.8) | 109 (50.5) | 0.103 |
|  | Yes | 720 (78.8) | 253 (78.6) |  | 724 (43.6) | 385 (39.4) |  | 258 (40.2) | 107 (49.5) |  |
| Feeding child(ren) | No | 230 (25.2) | 86 (26.7) | 0.923 | 1113 (67.0) | 693 (71.0) | 0.069 | 442 (69.0) | 147 (68.1) | 0.961 |
|  | Yes | 684 (74.8) | 236 (73.3) |  | 548 (33.0) | 283 (29.0) |  | 199 (31.0) | 69 (31.9) |  |
| Bathing child(ren) | No | 383 (41.9) | 130 (40.4) | 0.923 | 1085 (65.3) | 682 (69.9) | **0.046** | 385 (60.1) | 145 (67.1) | 0.217 |
|  | Yes | 531 (58.1) | 192 (59.6) |  | 576 (34.7) | 294 (30.1) |  | 256 (39.9) | 71 (32.9) |  |
| Reading bedtime stories | No | 438 (47.9) | 156 (48.4) | 1 | 1073 (64.6) | 730 (74.8) | **0.001** | 372 (58.0) | 148 (68.5) | 0.057 |
|  | Yes | 476 (52.1) | 166 (51.6) |  | 588 (35.4) | 246 (25.2) |  | 269 (42.0) | 68 (31.5) |  |
| Staying up in the night with child(ren) | No | 299 (32.7) | 82 (25.5) | 0.096 | 924 (55.6) | 577 (59.1) | 0.135 | 335 (52.3) | 121 (56.0) | 0.588 |
|  | Yes | 615 (67.3) | 240 (74.5) |  | 737 (44.4) | 399 (40.9) |  | 306 (47.7) | 95 (44.0) |  |
| Putting child(ren) to bed | No | 427 (46.7) | 132 (41.0) | 0.311 | 1129 (68.0) | 727 (74.5) | **0.002** | 391 (61.0) | 151 (69.9) | 0.105 |
|  | Yes | 487 (53.3) | 190 (59.0) |  | 532 (32.0) | 249 (25.5) |  | 250 (39.0) | 65 (30.1) |  |

Table S3. Frequencies and proportions of being primary caregivers in parenting-related activities among academic male respondents by academic partnership and parenting type (n=3,676).

| Activity | Primary | Lead | | | Dual | | | Satellite | | |
| --- | --- | --- | --- | --- | --- | --- | --- | --- | --- | --- |
|  |  | No | Yes | *p* | No | Yes | *p* | No | Yes | *p* |
| Coordinating playdates for child(ren) | No | 57 (64.8) | 18 (62.1) | 0.92 | 1259 (94.4) | 791 (95.3) | 0.535 | 1090 (98.9) | 289 (98.6) | 0.79 |
|  | Yes | 31 (35.2) | 11 (37.9) |  | 75 (5.6) | 39 (4.7) |  | 12 (1.1) | 4 (1.4) |  |
| Preparing birthday parties and other child events | No | 65 (73.9) | 26 (89.7) | 0.569 | 1290 (96.7) | 797 (96.0) | 0.55 | 1086 (98.5) | 287 (98.0) | 0.79 |
|  | Yes | 23 (26.1) | 3 (10.3) |  | 44 (3.3) | 33 (4.0) |  | 16 (1.5) | 6 (2.0) |  |
| Attending extracurricular events | No | 28 (31.8) | 13 (44.8) | 0.796 | 1077 (80.7) | 695 (83.7) | 0.225 | 1033 (93.7) | 279 (95.2) | 0.724 |
|  | Yes | 60 (68.2) | 16 (55.2) |  | 257 (19.3) | 135 (16.3) |  | 69 (6.3) | 14 (4.8) |  |
| Helping child(ren) with extracurricular events | No | 26 (29.5) | 13 (44.8) | 0.59 | 1100 (82.5) | 700 (84.3) | 0.447 | 1021 (92.6) | 278 (94.9) | 0.593 |
|  | Yes | 62 (70.5) | 16 (55.2) |  | 234 (17.5) | 130 (15.7) |  | 81 (7.4) | 15 (5.1) |  |
| Coaching a child's sporting team | No | 46 (52.3) | 21 (72.4) | 0.569 | 932 (69.9) | 588 (70.8) | 0.698 | 797 (72.3) | 228 (77.8) | 0.36 |
|  | Yes | 42 (47.7) | 8 (27.6) |  | 402 (30.1) | 242 (29.2) |  | 305 (27.7) | 65 (22.2) |  |
| Assisting with prayers/religious rituals | No | 73 (83.0) | 26 (89.7) | 0.92 | 1207 (90.5) | 770 (92.8) | 0.205 | 1021 (92.6) | 268 (91.5) | 0.79 |
|  | Yes | 15 (17.0) | 3 (10.3) |  | 127 (9.5) | 60 (7.2) |  | 81 (7.4) | 25 (8.5) |  |
| Preparing for holidays | No | 60 (68.2) | 18 (62.1) | 0.92 | 1222 (91.6) | 756 (91.1) | 0.706 | 1022 (92.7) | 271 (92.5) | 0.895 |
|  | Yes | 28 (31.8) | 11 (37.9) |  | 112 (8.4) | 74 (8.9) |  | 80 (7.3) | 22 (7.5) |  |
| Spending unstructured time with your child(ren) | No | 29 (33.0) | 9 (31.0) | 1 | 1129 (84.6) | 720 (86.7) | 0.396 | 1008 (91.5) | 270 (92.2) | 0.79 |
|  | Yes | 59 (67.0) | 20 (69.0) |  | 205 (15.4) | 110 (13.3) |  | 94 (8.5) | 23 (7.8) |  |
| Dropping of child(ren) at school/nursery | No | 21 (23.9) | 5 (17.2) | 0.92 | 737 (55.2) | 538 (64.8) | **0.002** | 819 (74.3) | 229 (78.2) | 0.593 |
|  | Yes | 67 (76.1) | 24 (82.8) |  | 597 (44.8) | 292 (35.2) |  | 283 (25.7) | 64 (21.8) |  |
| Picking up child(ren) from school/nursery | No | 21 (23.9) | 8 (27.6) | 0.92 | 934 (70.0) | 642 (77.3) | **0.004** | 996 (90.4) | 269 (91.8) | 0.79 |
|  | Yes | 67 (76.1) | 21 (72.4) |  | 400 (30.0) | 188 (22.7) |  | 106 (9.6) | 24 (8.2) |  |
| Shopping for household/child(ren) | No | 40 (45.5) | 11 (37.9) | 0.92 | 1083 (81.2) | 632 (76.1) | **0.038** | 967 (87.7) | 251 (85.7) | 0.724 |
|  | Yes | 48 (54.5) | 18 (62.1) |  | 251 (18.8) | 198 (23.9) |  | 135 (12.3) | 42 (14.3) |  |
| Taking child(ren) to doctor's appointments | No | 27 (30.7) | 8 (27.6) | 0.92 | 1112 (83.4) | 725 (87.3) | **0.043** | 1044 (94.7) | 280 (95.6) | 0.79 |
|  | Yes | 61 (69.3) | 21 (72.4) |  | 222 (16.6) | 105 (12.7) |  | 58 (5.3) | 13 (4.4) |  |
| Cooking and preparing weekday meals | No | 26 (29.5) | 11 (37.9) | 0.92 | 959 (71.9) | 584 (70.4) | 0.55 | 986 (89.5) | 255 (87.0) | 0.636 |
|  | Yes | 62 (70.5) | 18 (62.1) |  | 375 (28.1) | 246 (29.6) |  | 116 (10.5) | 38 (13.0) |  |
| Cooking and preparing weekend/holiday meals | No | 36 (40.9) | 13 (44.8) | 0.92 | 934 (70.0) | 559 (67.3) | 0.396 | 838 (76.0) | 226 (77.1) | 0.79 |
|  | Yes | 52 (59.1) | 16 (55.2) |  | 400 (30.0) | 271 (32.7) |  | 264 (24.0) | 67 (22.9) |  |
| Making weekday lunches | No | 43 (48.9) | 15 (51.7) | 0.92 | 1070 (80.2) | 643 (77.5) | 0.301 | 1011 (91.7) | 266 (90.8) | 0.79 |
|  | Yes | 45 (51.1) | 14 (48.3) |  | 264 (19.8) | 187 (22.5) |  | 91 (8.3) | 27 (9.2) |  |
| Preparing snacks for child(ren) | No | 45 (51.1) | 16 (55.2) | 0.92 | 1148 (86.1) | 709 (85.4) | 0.706 | 1063 (96.5) | 272 (92.8) | 0.199 |
|  | Yes | 43 (48.9) | 13 (44.8) |  | 186 (13.9) | 121 (14.6) |  | 39 (3.5) | 21 (7.2) |  |
| Feeding child(ren) | No | 39 (44.3) | 13 (44.8) | 1 | 1190 (89.2) | 750 (90.4) | 0.55 | 1080 (98.0) | 284 (96.9) | 0.724 |
|  | Yes | 49 (55.7) | 16 (55.2) |  | 144 (10.8) | 80 (9.6) |  | 22 (2.0) | 9 (3.1) |  |
| Bathing child(ren) | No | 58 (65.9) | 13 (44.8) | 0.569 | 1113 (83.4) | 706 (85.1) | 0.535 | 957 (86.8) | 262 (89.4) | 0.636 |
|  | Yes | 30 (34.1) | 16 (55.2) |  | 221 (16.6) | 124 (14.9) |  | 145 (13.2) | 31 (10.6) |  |
| Reading bedtime stories | No | 48 (54.5) | 10 (34.5) | 0.569 | 979 (73.4) | 649 (78.2) | **0.043** | 793 (72.0) | 227 (77.5) | 0.36 |
|  | Yes | 40 (45.5) | 19 (65.5) |  | 355 (26.6) | 181 (21.8) |  | 309 (28.0) | 66 (22.5) |  |
| Staying up in the night with child(ren) | No | 50 (56.8) | 14 (48.3) | 0.92 | 1071 (80.3) | 680 (81.9) | 0.535 | 957 (86.8) | 263 (89.8) | 0.593 |
|  | Yes | 38 (43.2) | 15 (51.7) |  | 263 (19.7) | 150 (18.1) |  | 145 (13.2) | 30 (10.2) |  |
| Putting child(ren) to bed | No | 46 (52.3) | 10 (34.5) | 0.569 | 1076 (80.7) | 708 (85.3) | **0.042** | 915 (83.0) | 260 (88.7) | 0.222 |
|  | Yes | 42 (47.7) | 19 (65.5) |  | 258 (19.3) | 122 (14.7) |  | 187 (17.0) | 33 (11.3) |  |

Table S4. Estimated coefficients from OLS models of productivity (P) and impact (TCS & MNCS).

|  | Full Sample | | | Two-Academics Sample | | |
| --- | --- | --- | --- | --- | --- | --- |
|  | log(P) | log(TCS) | log(MNCS) | log(P) | log(TCS) | log(MNCS) |
| (Intercept) | 2.77*** | 4.81*** | 0.05** | 2.79*** | 4.89*** | 0.15*** |
|  | [2.73, 2.81] (0.00) | [4.73, 4.88] (0.00) | [0.01, 0.09] (0.01) | [2.72, 2.86] (0.00) | [4.77, 5.02] (0.00) | [0.09, 0.21] (0.00) |
| ***Gender (ref. Women)*** |  |  |  |  |  |  |
| Men | 0.05* | 0.04 | 0.00 | 0.01 | -0.06 | -0.08* |
|  | [0.01, 0.10] (0.03) | [-0.05, 0.13] (0.43) | [-0.05, 0.06] (0.91) | [-0.06, 0.08] (0.80) | [-0.18, 0.06] (0.30) | [-0.16, -0.01] (0.03) |
| ***Parenting type (ref. Dual)*** | | | | | | |
| Lead | 0.00 | -0.01 | 0.01 | -0.12** | -0.26** | -0.19*** |
|  | [-0.05, 0.06] (0.86) | [-0.10, 0.09] (0.91) | [-0.06, 0.07] (0.86) | [-0.21, -0.03] (0.01) | [-0.43, -0.09] (0.00) | [-0.30, -0.08] (0.00) |
| Satellite | 0.09** | 0.08 | 0.08* | 0.00 | 0.07 | 0.06 |
|  | [0.03, 0.14] (0.00) | [-0.02, 0.18] (0.13) | [0.01, 0.14] (0.02) | [-0.12, 0.12] (1.00) | [-0.11, 0.24] (0.46) | [-0.05, 0.17] (0.30) |
| ***Partnership status (ref. Non-academic)*** | | | | | | |
| Academic | 0.05+ | 0.14** | 0.10*** |  |  |  |
|  | [-0.01, 0.10] (0.08) | [0.04, 0.24] (0.00) | [0.04, 0.17] (0.00) |  |  |  |
| Single | -0.17* | 0.03 | 0.05 |  |  |  |
|  | [-0.33, 0.00] (0.05) | [-0.28, 0.34] (0.85) | [-0.12, 0.21] (0.58) |  |  |  |
| ***Gender x Parenting type*** | | | | | | |
| Men x Lead | -0.12 | -0.35* | -0.11 | 0.02 | -0.01 | 0.05 |
|  | [-0.27, 0.03] (0.11) | [-0.65, -0.04] (0.03) | [-0.30, 0.08] (0.28) | [-0.28, 0.32] (0.87) | [-0.60, 0.59] (0.99) | [-0.29, 0.39] (0.77) |
| Men x Satellite | -0.02 | -0.02 | -0.05 | 0.03 | -0.02 | -0.02 |
|  | [-0.10, 0.06] (0.61) | [-0.17, 0.13] (0.78) | [-0.15, 0.04] (0.25) | [-0.12, 0.18] (0.70) | [-0.26, 0.23] (0.90) | [-0.18, 0.13] (0.77) |
| ***Gender x Partnership status*** | | | | | | |
| Men x Academic | -0.02 | -0.09 | -0.10* |  |  |  |
|  | [-0.10, 0.06] (0.64) | [-0.24, 0.05] (0.21) | [-0.19, -0.01] (0.03) |  |  |  |
| Men x Single | -0.02 | -0.21 | -0.11 |  |  |  |
|  | [-0.24, 0.21] (0.88) | [-0.62, 0.20] (0.31) | [-0.33, 0.11] (0.33) |  |  |  |
| ***Parenting type x Partnership status*** | | | | | | |
| Lead x Academic | -0.11* | -0.22* | -0.19** |  |  |  |
|  | [-0.20, -0.01] (0.04) | [-0.41, -0.03] (0.02) | [-0.31, -0.07] (0.00) |  |  |  |
| Satellite x Academic | -0.07 | 0.01 | -0.03 |  |  |  |
|  | [-0.20, 0.06] (0.28) | [-0.19, 0.21] (0.93) | [-0.16, 0.10] (0.64) |  |  |  |
| Lead x Single | 0.05 | -0.15 | -0.12 |  |  |  |
|  | [-0.13, 0.23] (0.60) | [-0.49, 0.19] (0.39) | [-0.31, 0.07] (0.22) |  |  |  |
| Satellite x Single | -0.13 | -0.52 | -0.30+ |  |  |  |
|  | [-0.50, 0.23] (0.47) | [-1.21, 0.17] (0.14) | [-0.63, 0.04] (0.08) |  |  |  |
| ***Gender x Parenting type x Partnership status*** | | | | | | |
| Men x Lead x Academic | 0.08 | 0.28 | 0.11 |  |  |  |
|  | [-0.24, 0.41] (0.61) | [-0.37, 0.92] (0.40) | [-0.28, 0.51] (0.57) |  |  |  |
| Men x Satellite x Academic | 0.04 | 0.00 | 0.05 |  |  |  |
|  | [-0.13, 0.21] (0.62) | [-0.28, 0.28] (0.98) | [-0.13, 0.23] (0.58) |  |  |  |
| Men x Lead x Single | 0.12 | 0.31 | 0.11 |  |  |  |
|  | [-0.25, 0.48] (0.54) | [-0.37, 0.99] (0.37) | [-0.27, 0.49] (0.58) |  |  |  |
| Men x Satellite x Single | 0.09 | 0.43 | 0.24 |  |  |  |
|  | [-0.37, 0.55] (0.71) | [-0.44, 1.30] (0.33) | [-0.21, 0.69] (0.30) |  |  |  |
| ***Academic age (centered)*** | | | | | | |
| 1st degree | 0.14*** | 0.21*** | 0.03*** | 0.13*** | 0.21*** | 0.03*** |
|  | [0.13, 0.14] (0.00) | [0.21, 0.22] (0.00) | [0.03, 0.03] (0.00) | [0.13, 0.14] (0.00) | [0.21, 0.22] (0.00) | [0.02, 0.03] (0.00) |
| 2nd degree | 0.00*** | -0.01*** | 0.00*** | 0.00*** | -0.01*** | 0.00*** |
|  | [0.00, 0.00] (0.00) | [-0.01, -0.01] (0.00) | [0.00, 0.00] (0.00) | [0.00, 0.00] (0.00) | [-0.01, 0.00] (0.00) | [0.00, 0.00] (0.00) |
| ***Number of children*** |  |  |  |  |  |  |
| 1 | 0.03+ | 0.02 | 0.01 | 0.01 | 0.05 | 0.05 |
|  | [0.00, 0.07] (0.05) | [-0.05, 0.08] (0.60) | [-0.03, 0.05] (0.49) | [-0.05, 0.08] (0.67) | [-0.07, 0.16] (0.44) | [-0.02, 0.13] (0.19) |
| 3 | -0.03 | -0.13*** | -0.07** | -0.05 | -0.21** | -0.13** |
|  | [-0.07, 0.01] (0.14) | [-0.21, -0.06] (0.00) | [-0.12, -0.03] (0.00) | [-0.14, 0.03] (0.20) | [-0.35, -0.07] (0.00) | [-0.21, -0.04] (0.00) |
| 4 and more | -0.08* | -0.22*** | -0.12*** | 0.01 | -0.18 | -0.12+ |
|  | [-0.14, -0.01] (0.02) | [-0.34, -0.10] (0.00) | [-0.20, -0.05] (0.00) | [-0.12, 0.14] (0.92) | [-0.42, 0.07] (0.16) | [-0.26, 0.01] (0.07) |
| Academic employment [No] | -0.16*** | -0.11** | -0.03 |  |  |  |
|  | [-0.20, -0.11] (0.00) | [-0.18, -0.03] (0.01) | [-0.08, 0.02] (0.21) |  |  |  |
| Doctoral degree [No] | -0.26*** | -0.32*** | -0.08* | -0.19* | -0.18 | 0.01 |
|  | [-0.32, -0.20] (0.00) | [-0.44, -0.21] (0.00) | [-0.15, -0.01] (0.03) | [-0.35, -0.03] (0.02) | [-0.46, 0.10] (0.21) | [-0.17, 0.19] (0.90) |
| ***Discipline (ref. Social sciences)*** | | | | | | |
| Arts and humanities | -0.30*** | -1.80*** |  | -0.32*** | -1.88*** |  |
|  | [-0.36, -0.25] (0.00) | [-1.93, -1.66] (0.00) |  | [-0.42, -0.21] (0.00) | [-2.11, -1.64] (0.00) |  |
| Health sciences | 0.59*** | 0.76*** |  | 0.65*** | 0.86*** |  |
|  | [0.56, 0.63] (0.00) | [0.69, 0.82] (0.00) |  | [0.58, 0.73] (0.00) | [0.73, 0.99] (0.00) |  |
| Natural sciences | 0.25*** | 0.36*** |  | 0.29*** | 0.42*** |  |
|  | [0.21, 0.28] (0.00) | [0.29, 0.43] (0.00) |  | [0.21, 0.36] (0.00) | [0.29, 0.55] (0.00) |  |
| Num.Obs. | 10013 | 10013 | 10013 | 2568 | 2568 | 2568 |
| R2 | 0.704 | 0.653 | 0.079 | 0.688 | 0.670 | 0.072 |
| R2 Adj. | 0.703 | 0.652 | 0.077 | 0.686 | 0.668 | 0.068 |
| AIC | 21822.4 | 33705.1 | 24261.9 | 5585.3 | 8517.6 | 6119.4 |
| BIC | 22031.5 | 33914.3 | 24449.4 | 5678.9 | 8611.2 | 6195.5 |
| Log.Lik. | -10882.206 | -16823.572 | -12104.953 | -2776.626 | -4242.801 | -3046.708 |
| F | 877.652 | 696.898 | 35.687 | 402.196 | 370.269 | 18.028 |
| Std. Errors | Robust | Robust | Robust | Robust | Robust | Robust |

Note: Numbers in the squared brackets are confidence intervals; numbers in the parentheses are *p* values.

+ *p*<0.1, * *p*<0.05, ** *p*<0.01, *** *p*<0.001

Table S5. Estimated coefficients from OLS models on productivity (P) for the US-only and non-US samples.

|  | US-only | Non-US |
| --- | --- | --- |
|  | log(P) | log(P) |
| (Intercept) | 2.60*** | 2.76*** |
|  | [2.53, 2.67] (0.00) | [2.67, 2.84] (0.00) |
| ***Gender (ref. Women)*** |  |  |
| Men | 0.15*** | 0.05 |
|  | [0.06, 0.24] (0.00) | [-0.04, 0.14] (0.31) |
| ***Parenting type (ref. Dual)*** |  |  |
| Lead | 0.00 | 0.01 |
|  | [-0.08, 0.08] (0.94) | [-0.06, 0.09] (0.69) |
| Satellite | 0.10* | 0.06 |
|  | [0.02, 0.18] (0.02) | [-0.03, 0.15] (0.22) |
| ***Partnership status (ref. Non-academic)*** |  |  |
| Academic | 0.07+ | 0.02 |
|  | [-0.01, 0.15] (0.08) | [-0.06, 0.10] (0.65) |
| Single | -0.13 | -0.16 |
|  | [-0.35, 0.09] (0.24) | [-0.36, 0.05] (0.13) |
| ***Parental leave length (ref. No leave)*** |  |  |
| Less than 1 month | 0.24** | -0.07 |
|  | [0.08, 0.39] (0.00) | [-0.36, 0.21] (0.61) |
| At least 1 but less than 3 months | 0.24*** | 0.18** |
|  | [0.17, 0.30] (0.00) | [0.06, 0.29] (0.00) |
| 3 to 6 months | 0.16*** | 0.10** |
|  | [0.09, 0.23] (0.00) | [0.03, 0.18] (0.01) |
| Greater than 6 but less than 12 months | 0.00 | 0.10** |
|  | [-0.17, 0.17] (0.97) | [0.03, 0.18] (0.01) |
| 12 months and more | 0.00 | -0.04 |
|  | [-0.52, 0.51] (0.99) | [-0.15, 0.06] (0.44) |
| ***Gender x Parenting type*** |  |  |
| Men × Lead | -0.09 | -0.14 |
|  | [-0.35, 0.17] (0.49) | [-0.35, 0.07] (0.19) |
| Men × Satellite | -0.07 | 0.06 |
|  | [-0.19, 0.04] (0.23) | [-0.06, 0.17] (0.32) |
| Men × Academic | -0.07 | 0.05 |
|  | [-0.18, 0.05] (0.26) | [-0.07, 0.16] (0.41) |
| Men × Single | 0.01 | -0.04 |
|  | [-0.29, 0.32] (0.93) | [-0.32, 0.23] (0.75) |
| ***Parenting type x Partnership status*** |  |  |
| Lead × Academic | -0.08 | -0.10 |
|  | [-0.23, 0.07] (0.29) | [-0.24, 0.04] (0.14) |
| Satellite × Academic | -0.06 | -0.11 |
|  | [-0.22, 0.10] (0.45) | [-0.30, 0.07] (0.24) |
| Lead × Single | 0.02 | 0.05 |
|  | [-0.23, 0.27] (0.88) | [-0.19, 0.28] (0.69) |
| Satellite × Single | 0.13 | -0.51* |
|  | [-0.36, 0.62] (0.60) | [-1.01, -0.01] (0.05) |
| ***Gender x Parental leave length*** |  |  |
| Men × Less than 1 month | 0.05 | 0.17 |
|  | [-0.13, 0.23] (0.61) | [-0.12, 0.47] (0.24) |
| Men × At least 1 but less than 3 months | -0.04 | -0.04 |
|  | [-0.18, 0.11] (0.63) | [-0.20, 0.12] (0.62) |
| Men × 3 to 6 months | -0.07 | 0.00 |
|  | [-0.21, 0.08] (0.38) | [-0.13, 0.13] (0.98) |
| Men × Greater than 6 but less than 12 months | -0.16 | 0.01 |
|  | [-0.83, 0.51] (0.64) | [-0.16, 0.19] (0.89) |
| Men × 12 months and more | 0.66 | -0.24 |
|  | [-0.87, 2.19] (0.40) | [-0.57, 0.10] (0.16) |
| ***Gender x Parenting type x Partnership status*** |  |  |
| Men × Lead × Academic | 0.27 | -0.17 |
|  | [-0.19, 0.74] (0.25) | [-0.63, 0.28] (0.46) |
| Men × Satellite × Academic | 0.13 | -0.02 |
|  | [-0.09, 0.36] (0.26) | [-0.26, 0.21] (0.85) |
| Men × Lead × Single | 0.10 | 0.15 |
|  | [-0.41, 0.61] (0.69) | [-0.30, 0.59] (0.53) |
| Men × Satellite × Single | -0.31 | 0.50+ |
|  | [-0.99, 0.37] (0.37) | [-0.09, 1.09] (0.10) |
| ***Academic age (centered)*** |  |  |
| 1^st^ degree | 0.14*** | 0.14*** |
|  | [0.13, 0.14] (0.00) | [0.14, 0.14] (0.00) |
| 2^nd^ degree | 0.00*** | 0.00*** |
|  | [0.00, 0.00] (0.00) | [0.00, 0.00] (0.00) |
| ***Number of children (ref. 2)*** |  |  |
| 1 | 0.01 | 0.05* |
|  | [-0.04, 0.06] (0.58) | [0.00, 0.10] (0.03) |
| 3 | -0.04 | -0.03 |
|  | [-0.10, 0.02] (0.19) | [-0.08, 0.02] (0.27) |
| 4 and more | -0.01 | -0.12* |
|  | [-0.10, 0.08] (0.77) | [-0.21, -0.03] (0.01) |
| Academic employment [No] | -0.12*** | -0.20*** |
|  | [-0.19, -0.06] (0.00) | [-0.26, -0.15] (0.00) |
| Doctoral degree [No] | -0.20*** | -0.31*** |
|  | [-0.29, -0.10] (0.00) | [-0.39, -0.23] (0.00) |
| ***Discipline (ref. Social sciences)*** |  |  |
| Arts & humanities | -0.33*** | -0.27*** |
|  | [-0.42, -0.23] (0.00) | [-0.36, -0.18] (0.00) |
| Health sciences | 0.57*** | 0.55*** |
|  | [0.51, 0.62] (0.00) | [0.50, 0.61] (0.00) |
| Natural sciences | 0.23*** | 0.22*** |
|  | [0.18, 0.29] (0.00) | [0.17, 0.27] (0.00) |
| Num.Obs. | 5010 | 5003 |
| R2 | 0.691 | 0.726 |
| R2 Adj. | 0.689 | 0.724 |
| AIC | 11140.6 | 10583.2 |
| BIC | 11394.8 | 10837.4 |
| Log.Lik. | -5531.300 | -5252.582 |
| F | 301.175 | 354.891 |

Note: Numbers in the squared brackets are confidence intervals; numbers in the parentheses are *p* values.

+ *p*<0.1, * *p*<0.05, ** *p*< 0.01, *** *p*<0.001

Table S6. Number and percentage of respondents, of sampled authors, and population, by main country of affiliation.

| Country | Declared in Survey | |  | Respondents | | |  | Sample | |  | Population |
| --- | --- | --- | --- | --- | --- | --- | --- | --- | --- | --- | --- |
|  | N | % of respondents |  | N | % of  sample | % of  population |  | N | % of  population |  |  |
| United States | 5,197 | 49.76% |  | 4,828 | 1.64% | 0.94% |  | 295,236 | 57.41% |  | 514,245 |
| United Kingdom | 972 | 9.31% |  | 884 | 1.19% | 0.68% |  | 74,298 | 57.31% |  | 129,633 |
| Canada | 642 | 6.15% |  | 602 | 1.48% | 0.85% |  | 40,614 | 57.18% |  | 71,033 |
| Australia | 421 | 4.03% |  | 416 | 1.23% | 0.70% |  | 33,791 | 57.18% |  | 59,098 |
| Germany | 345 | 3.30% |  | 307 | 0.45% | 0.26% |  | 68,710 | 57.22% |  | 120,074 |
| France | 233 | 2.23% |  | 223 | 0.44% | 0.25% |  | 50,798 | 57.05% |  | 89,044 |
| Netherlands | 218 | 2.09% |  | 214 | 0.88% | 0.50% |  | 24,389 | 57.02% |  | 42,776 |
| Italy | 204 | 1.95% |  | 193 | 0.45% | 0.26% |  | 42,811 | 57.31% |  | 74,706 |
| Spain | 195 | 1.87% |  | 193 | 0.46% | 0.25% |  | 42,075 | 55.13% |  | 76,316 |
| Sweden | 171 | 1.64% |  | 164 | 0.97% | 0.55% |  | 16,906 | 56.42% |  | 29,966 |
| Switzerland | 127 | 1.22% |  | 121 | 0.84% | 0.48% |  | 14,334 | 56.70% |  | 25,282 |
| New Zealand | 121 | 1.16% |  | 118 | 2.06% | 1.18% |  | 5,717 | 57.34% |  | 9,970 |
| Norway | 120 | 1.15% |  | 114 | 1.29% | 0.74% |  | 8,804 | 57.53% |  | 15,304 |
| Finland | 93 | 0.89% |  | 89 | 0.96% | 0.55% |  | 9,294 | 57.09% |  | 16,280 |
| Portugal | 84 | 0.80% |  | 83 | 0.92% | 0.53% |  | 9,010 | 57.68% |  | 15,622 |
| Belgium | 80 | 0.77% |  | 74 | 0.60% | 0.34% |  | 12,367 | 57.10% |  | 21,658 |
| Poland | 78 | 0.75% |  | 76 | 0.41% | 0.23% |  | 18,727 | 56.51% |  | 33,138 |
| Denmark | 77 | 0.74% |  | 72 | 0.73% | 0.42% |  | 9,859 | 57.99% |  | 17,002 |
| Greece | 72 | 0.69% |  | 74 | 0.91% | 0.51% |  | 8,113 | 55.96% |  | 14,498 |
| Brazil | 64 | 0.61% |  | 63 | 0.14% | 0.08% |  | 46,330 | 56.94% |  | 81,362 |
| South Africa | 63 | 0.60% |  | 62 | 0.87% | 0.50% |  | 7,090 | 57.11% |  | 12,415 |
| Austria | 63 | 0.60% |  | 56 | 0.68% | 0.39% |  | 8,288 | 57.35% |  | 14,451 |
| Ireland | 46 | 0.44% |  | 46 | 0.89% | 0.51% |  | 5,173 | 56.95% |  | 9,083 |
| Turkey | 46 | 0.44% |  | 44 | 0.18% | 0.10% |  | 24,743 | 55.37% |  | 44,689 |
| Czech Republic | 40 | 0.38% |  | 37 | 0.44% | 0.25% |  | 8,418 | 57.55% |  | 14,627 |
| Mexico | 40 | 0.38% |  | 39 | 0.37% | 0.20% |  | 10,612 | 54.41% |  | 19,504 |
| Israel | 39 | 0.37% |  | 34 | 0.41% | 0.23% |  | 8,232 | 55.66% |  | 14,790 |
| India | 38 | 0.36% |  | 35 | 0.08% | 0.04% |  | 41,918 | 53.76% |  | 77,969 |
| Croatia | 37 | 0.35% |  | 37 | 0.95% | 0.54% |  | 3,878 | 56.99% |  | 6,805 |
| Russia | 34 | 0.33% |  | 36 | 0.16% | 0.09% |  | 21,846 | 52.23% |  | 41,828 |
| Slovenia | 29 | 0.28% |  | 28 | 0.91% | 0.51% |  | 3,064 | 55.60% |  | 5,511 |
| Japan | 28 | 0.27% |  | 25 | 0.04% | 0.02% |  | 59,415 | 56.98% |  | 104,270 |
| Hungary | 26 | 0.25% |  | 23 | 0.52% | 0.28% |  | 4,462 | 53.80% |  | 8,294 |
| Serbia | 24 | 0.23% |  | 24 | 0.52% | 0.28% |  | 4,650 | 53.90% |  | 8,627 |
| Singapore | 23 | 0.22% |  | 21 | 0.35% | 0.19% |  | 5,973 | 55.44% |  | 10,774 |
| Argentina | 22 | 0.21% |  | 21 | 0.26% | 0.15% |  | 7,998 | 55.77% |  | 14,342 |
| China | 21 | 0.20% |  | 32 | 0.02% | 0.01% |  | 153,786 | 58.44% |  | 263,159 |
| Others (N = 162) | 312 | 2.99% |  | 276 | 0.18% | 0.10% |  | 152,042 | 54.80% |  | 277,448 |
| *Unknown* | *-* | *-* |  | *661* | *0.49%* | *0.28%* |  | *136,229* | *57.90%* |  | *235,279* |
| All | 10,445 | 100.00% |  | 10,445 | 0.70% | 0.40% |  | ####### | 56.80% |  | ####### |

Table S7. Number and percentage of respondents, of sampled authors, and population, by main discipline.

| Discipline | Declared in Survey | |  | Respondents | | |  | Sample | |  | Population |
| --- | --- | --- | --- | --- | --- | --- | --- | --- | --- | --- | --- |
|  | N | % of respondents |  | N | % of  sample | % of  population |  | N | % of  population |  |  |
| Social Sciences | 1,686 | 16.14% |  | 1,146 | 1.61% | 0.93% |  | 71,010 | 57.46% |  | 123,591 |
| Clinical Medicine | 1,115 | 10.67% |  | 2,452 | 0.64% | 0.36% |  | 382,307 | 56.28% |  | 679,340 |
| Biomedical Research | 958 | 9.17% |  | 891 | 0.65% | 0.37% |  | 137,295 | 56.79% |  | 241,764 |
| Psychology | 953 | 9.12% |  | 656 | 1.97% | 1.13% |  | 33,253 | 57.40% |  | 57,931 |
| Engineering | 899 | 8.61% |  | 651 | 0.26% | 0.15% |  | 252,053 | 57.13% |  | 441,215 |
| Biology | 893 | 8.55% |  | 647 | 0.56% | 0.32% |  | 115,371 | 56.74% |  | 203,324 |
| Health | 815 | 7.80% |  | 522 | 1.29% | 0.74% |  | 40,405 | 57.50% |  | 70,272 |
| Humanities | 639 | 6.12% |  | 323 | 1.07% | 0.61% |  | 30,164 | 57.30% |  | 52,646 |
| Physics | 444 | 4.25% |  | 365 | 0.34% | 0.19% |  | 108,623 | 56.86% |  | 191,045 |
| Earth and Space | 438 | 4.19% |  | 631 | 0.63% | 0.36% |  | 100,225 | 57.24% |  | 175,093 |
| Professional Fields | 402 | 3.85% |  | 913 | 1.53% | 0.88% |  | 59,503 | 57.57% |  | 103,360 |
| Mathematics | 352 | 3.37% |  | 243 | 0.44% | 0.25% |  | 54,973 | 57.58% |  | 95,469 |
| Chemistry | 340 | 3.26% |  | 312 | 0.32% | 0.18% |  | 98,166 | 55.49% |  | 176,923 |
| Arts | 53 | 0.51% |  | 32 | 0.81% | 0.47% |  | 3,959 | 57.88% |  | 6,840 |
| Other / Unknown | 458 | 4.38% |  | 661 | 5.21% | 3.00% |  | 12,693 | 57.54% |  | 22,059 |
| All | 10,445 | 100.00% |  | 10,445 | 0.70% | 0.40% |  | 1,500,000 | 56.80% |  | 2,640,872 |

Table S8. Number and percentage of respondents, of sampled authors, and population, by perceived gender.

Table S9. Descriptive Statistics.

| Variable |  | Women | Men | Total |
| --- | --- | --- | --- | --- |
| Number of papers | Mean (SD) | 28.9 (52.8) | 41.5 (67.7) | 34.2 (59.9) |
| MNCS | Mean (SD) | 1.3 (1.2) | 1.4 (1.6) | 1.3 (1.4) |
| Academic age | Mean (SD) | 12.1 (8.3) | 15.3 (9.8) | 13.4 (9.1) |
| Parenting type | Lead | 1847 (30.6) | 174 (3.9) | 2021 (19.3) |
|  | Dual | 3132 (52.0) | 2526 (57.2) | 5658 (54.2) |
|  | Satellite | 1049 (17.4) | 1717 (38.9) | 2766 (26.5) |
| Partnered relationship | No | 508 (8.4) | 209 (4.7) | 717 (6.9) |
|  | Yes | 5520 (91.6) | 4208 (95.3) | 9728 (93.1) |
| Number of children | 1 | 1720 (28.5) | 982 (22.2) | 2702 (25.9) |
|  | 2 | 3233 (53.6) | 2239 (50.7) | 5472 (52.4) |
|  | 3 | 887 (14.7) | 827 (18.7) | 1714 (16.4) |
|  | 4+ | 188 (3.1) | 369 (8.4) | 557 (5.3) |
| Length of parental leaves | No leave | 1454 (24.1) | 2753 (62.3) | 4207 (40.3) |
|  | Less than 1 month | 132 (2.2) | 872 (19.7) | 1004 (9.6) |
|  | Less than 3 months | 1323 (21.9) | 336 (7.6) | 1659 (15.9) |
|  | 3–6 months | 1802 (29.9) | 343 (7.8) | 2145 (20.5) |
|  | Greater than 6, but less than 12 months | 1038 (17.2) | 92 (2.1) | 1130 (10.8) |
|  | More than 12 months | 279 (4.6) | 21 (0.5) | 300 (2.9) |
| Domain | Arts and Humanities | 430 (7.1) | 281 (6.4) | 711 (6.8) |
|  | Health Sciences | 1971 (32.7) | 1069 (24.2) | 3040 (29.1) |
|  | Natural Sciences | 1538 (25.5) | 1896 (42.9) | 3434 (32.9) |
|  | Social Sciences | 2089 (34.7) | 1171 (26.5) | 3260 (31.2) |
| Doctoral degree | No | 445 (7.4) | 209 (4.7) | 654 (6.3) |
|  | Yes | 5583 (92.6) | 4208 (95.3) | 9791 (93.7) |
| Academic employment | No | 858 (14.2) | 557 (12.6) | 1415 (13.5) |
|  | Yes | 5170 (85.8) | 3860 (87.4) | 9030 (86.5) |
| Partner in academic employment | No | 3905 (70.7) | 2972 (70.6) | 6877 (70.7) |
|  | Yes | 1615 (29.3) | 1236 (29.4) | 2851 (29.3) |
| Two-academics household | No | 4006 (72.6) | 3056 (72.6) | 7062 (72.6) |
|  | Yes | 1514 (27.4) | 1152 (27.4) | 2666 (27.4) |

Table S10. Models and types of parenting.

| Parenting type | Definition |
| --- | --- |
| Lead parent | Any relationship arrangement AND “the primary caregiver” |
| Satellite parents | Any relationship arrangement AND have a “partner who is a primary caregiver”; or “the majority of childcare is performed by a non-parental caregiver(s)/other” |
| Dual parents | Any relationship arrangement AND have “share equal parenting roles with a partner”; AND “share equal parenting roles with non-parental caregivers(s)/other” |

Table S11. Employment sectors among female respondents and their partners (N=5,520).

| Partner  Respondent | Academic/  teaching | Academic/  research | Academic/  teaching & research | Government | Private/  research | Private/  non-research | Retired | Not employed | Other | Total |
| --- | --- | --- | --- | --- | --- | --- | --- | --- | --- | --- |
| Academic/  teaching | 41 (0.7%) | 11 (0.2%) | 23 (0.4%) | 19 (0.3%) | 3 (0.1%) | 118 (2.1%) | 14 (0.3%) | 15 (0.3%) | 69 (1.2%) | **313 (5.7%)** |
| Academic/  research | 44 (0.8%) | 163 (3.0%) | 96 (1.7%) | 81 (1.5%) | 47 (0.9%) | 370 (6.7%) | 18 (0.3%) | 28 (0.5%) | 168 (3.0%) | **1015 (18.4%)** |
| Academic/  teaching & research | 184 (3.3%) | 132 (2.4%) | 820 (14.9%) | 254 (4.6%) | 113 (2.0%) | 1162 (21.1%) | 69 (1.2%) | 137 (2.5%) | 531 (9.6%) | **3402 (61.6%)** |
| Government | 4 (0.1%) | 6 (0.1%) | 14 (0.3%) | 72 (1.3%) | 12 (0.2%) | 98 (1.8%) | 9 (0.2%) | 14 (0.3%) | 29 (0.5%) | **258 (4.7%)** |
| Private/  research | 1 (0.0%) | 5 (0.1%) | 9 (0.2%) | 13 (0.2%) | 19 (0.3%) | 68 (1.2%) | 1 (0.0%) | 4 (0.1%) | 19 (0.3%) | **139 (2.5%)** |
| Private/  non-research | 2 (0.0%) | 2 (0.0%) | 6 (0.1%) | 6 (0.1%) | 1 (0.0%) | 47 (0.9%) | 3 (0.1%) | 6 (0.1%) | 4 (0.1%) | **77 (1.4%)** |
| Retired | 3 (0.1%) | 0 (0.0%) | 2 (0.0%) | 3 (0.1%) | 1 (0.0%) | 4 (0.1%) | 30 (0.5%) | 2 (0.0%) | 5 (0.1%) | **50 (0.9%)** |
| Not employed | 0 (0.0%) | 1 (0.0%) | 2 (0.0%) | 4 (0.1%) | 0 (0.0%) | 6 (0.1%) | 1 (0.0%) | 0 (0.0%) | 2 (0.0%) | **16 (0.3%)** |
| Other | 6 (0.1%) | 14 (0.3%) | 24 (0.4%) | 21 (0.4%) | 3 (0.1%) | 89 (1.6%) | 8 (0.1%) | 6 (0.1%) | 79 (1.4%) | **250 (4.5%)** |
| Total | **285 (5.2%)** | **334 (6.1%)** | **996 (18.0%)** | **473 (8.6%)** | **199 (3.6%)** | **1962 (35.5%)** | **153 (2.8%)** | **212 (3.8%)** | **906 (16.4%)** | **5520 (100.0%)** |

Table S12. Employments sector among male respondents and their partners (N=4,208).

| Partner  Respondent | Academic/  teaching | Academic/  research | Academic/  teaching & research | Government | Private/  research | Private/  non-research | Retired | Not employed | Other | Total |
| --- | --- | --- | --- | --- | --- | --- | --- | --- | --- | --- |
| Academic/  teaching | 38 (0.9%) | 4 (0.1%) | 12 (0.3%) | 12 (0.3%) | 4 (0.1%) | 48 (1.1%) | 7 (0.2%) | 27 (0.6%) | 30 (0.7%) | **182 (4.3%)** |
| Academic/  research | 32 (0.8%) | 112 (2.7%) | 50 (1.2%) | 71 (1.7%) | 21 (0.5%) | 157 (3.7%) | 10 (0.2%) | 83 (2.0%) | 108 (2.6%) | **644 (15.3%)** |
| Academic/  teaching & research | 247 (5.9%) | 157 (3.7%) | 500 (11.9%) | 263 (6.2%) | 67 (1.6%) | 697 (16.6%) | 65 (1.5%) | 367 (8.7%) | 487 (11.6%) | **2850 (67.7%)** |
| Government | 16 (0.4%) | 7 (0.2%) | 5 (0.1%) | 43 (1.0%) | 10 (0.2%) | 45 (1.1%) | 3 (0.1%) | 38 (0.9%) | 28 (0.7%) | **195 (4.6%)** |
| Private/  research | 11 (0.3%) | 3 (0.1%) | 8 (0.2%) | 3 (0.1%) | 11 (0.3%) | 32 (0.8%) | 7 (0.2%) | 21 (0.5%) | 8 (0.2%) | **104 (2.5%)** |
| Private/  non-research | 6 (0.1%) | 1 (0.0%) | 4 (0.1%) | 2 (0.0%) | 1 (0.0%) | 21 (0.5%) | 2 (0.0%) | 9 (0.2%) | 3 (0.1%) | **49 (1.2%)** |
| Retired | 1 (0.0%) | 1 (0.0%) | 4 (0.1%) | 1 (0.0%) | 0 (0.0%) | 9 (0.2%) | 63 (1.5%) | 6 (0.1%) | 5 (0.1%) | **90 (2.1%)** |
| Not employed | 0 (0.0%) | 0 (0.0%) | 1 (0.0%) | 0 (0.0%) | 0 (0.0%) | 1 (0.0%) | 0 (0.0%) | 1 (0.0%) | 0 (0.0%) | **3 (0.1%)** |
| Other | 8 (0.2%) | 2 (0.0%) | 6 (0.1%) | 6 (0.1%) | 1 (0.0%) | 12 (0.3%) | 1 (0.0%) | 17 (0.4%) | 38 (0.9%) | **91 (2.2%)** |
| Total | **359 (8.5%)** | **287 (6.8%)** | **590 (14.0%)** | **401 (9.5%)** | **115 (2.7%)** | **1022 (24.3%)** | **158 (3.8%)** | **569 (13.5%)** | **707 (16.8%)** | **4208 (100.0%)** |

Table S13. Parenting type by gender in the US and the non-US samples.

|  | Gender | Lead | Dual | Satellite | Total |
| --- | --- | --- | --- | --- | --- |
| Non-US | Women | 961 (34.9%) | 1397 (50.7%) | 395 (14.3%) | 2753 |
|  | Men | 88 (3.9%) | 1291 (57.4%) | 871 (38.7%) | 2250 |
|  | Total | 1049 (21.0%) | 2688 (53.7%) | 1266 (25.3%) | 5003 |
| US | Women | 799 (26.6%) | 1590 (52.9%) | 614 (20.4%) | 3003 |
|  | Men | 75 (3.7%) | 1152 (57.4%) | 780 (38.9%) | 2007 |
|  | Total | 874 (17.4%) | 2742 (54.7%) | 1394 (27.8%) | 5010 |

Table S14. Parental leave length by gender in the US and the non-US samples.

| Sample | Gender | No leave | <1 mo | <3 mo | 3–6 mo | >6 but <12 mo | >=12 mo | Total |
| --- | --- | --- | --- | --- | --- | --- | --- | --- |
| Non-US | Women | 527 (19.1%) | 24  (0.9%) | 188 (6.8%) | **843 (30.6%)** | **923 (33.5%)** | 248 (9.0%) | 2753 |
|  | Men | 1189 (52.8%) | **572 (25.4%)** | 195 (8.7%) | 194 (8.6%) | 81  (3.6%) | 19  (0.8%) | 2250 |
|  | Total | 1716 (34.3%) | 596 (11.9%) | 383 (7.7%) | 1037 (20.7%) | 1004 (20.1%) | 267 (5.3%) | 5003 |
| US | Women | 840 (28.0%) | 98  (3.3%) | **1094 (36.4%)** | **885 (29.5%)** | 78  (2.6%) | 8  (0.3%) | 3003 |
|  | Men | 1451 (72.3%) | **279 (13.9%)** | 132 (6.6%) | 139 (6.9%) | 5  (0.2%) | 1  (0.0%) | 2007 |
|  | Total | 2291 (45.7%) | 377 (7.5%) | 1226 (24.5%) | 1024 (20.4%) | 83  (1.7%) | 9  (0.2%) | 5010 |

Table S15. Estimated coefficients from OLS models on productivity (P) and impact (TCS) of different sample sizes and predictors.

|  | log(N Papers) | | log(TCS) | | |
| --- | --- | --- | --- | --- | --- |
|  | n=10,445 | n=10,013 | n=10,445 | n=10,013 | n=10,013* |
| (Intercept) | 2.77*** | 2.77*** | 4.81*** | 4.81*** | 4.58*** |
|  | [2.73, 2.81] (0.00) | [2.73, 2.81] (0.00) | [4.74, 4.88] (0.00) | [4.73, 4.88] (0.00) | [4.50, 4.67] (0.00) |
| ***Gender (ref. Women)*** |  |  |  |  |  |
| Men | 0.05* | 0.05* | 0.05 | 0.04 | 0.03 |
|  | [0.01, 0.10] (0.03) | [0.01, 0.10] (0.03) | [-0.04, 0.13] (0.29) | [-0.05, 0.13] (0.43) | [-0.05, 0.11] (0.45) |
| ***Parenting type (ref. Dual)*** |  |  |  |  |  |
| Lead | 0.01 | 0.00 | 0.01 | -0.01 | -0.01 |
|  | [-0.04, 0.06] (0.69) | [-0.05, 0.06] (0.86) | [-0.09, 0.10] (0.90) | [-0.10, 0.09] (0.91) | [-0.10, 0.08] (0.79) |
| Satellite | 0.08** | 0.09** | 0.06 | 0.08 | 0.08 |
|  | [0.03, 0.14] (0.00) | [0.03, 0.14] (0.00) | [-0.04, 0.16] (0.21) | [-0.02, 0.18] (0.13) | [-0.02, 0.17] (0.11) |
| ***Partnership status (ref. Non-academic)*** | | | | | |
| Academic | 0.04 | 0.05+ | 0.12* | 0.14** | 0.12* |
|  | [-0.01, 0.10] (0.12) | [-0.01, 0.10] (0.08) | [0.03, 0.21] (0.01) | [0.04, 0.24] (0.00) | [0.03, 0.21] (0.01) |
| Single | -0.14+ | -0.17* | 0.07 | 0.03 | 0.10 |
|  | [-0.30, 0.02] (0.09) | [-0.33, 0.00] (0.05) | [-0.21, 0.35] (0.63) | [-0.28, 0.34] (0.85) | [-0.18, 0.37] (0.49) |
| ***Gender x Parenting type*** |  |  |  |  |  |
| Men x Lead | -0.13+ | -0.12 | -0.40** | -0.35* | -0.29* |
|  | [-0.28, 0.02] (0.09) | [-0.27, 0.03] (0.11) | [-0.69, -0.10] (0.01) | [-0.65, -0.04] (0.03) | [-0.57, -0.02] (0.03) |
| Men x Satellite | -0.03 | -0.02 | -0.04 | -0.02 | -0.07 |
|  | [-0.11, 0.05] (0.48) | [-0.10, 0.06] (0.61) | [-0.19, 0.10] (0.54) | [-0.17, 0.13] (0.78) | [-0.21, 0.06] (0.27) |
| ***Gender x Partnership status*** |  |  |  |  |  |
| Men x Academic | -0.02 | -0.02 | -0.10 | -0.09 | -0.10 |
|  | [-0.10, 0.06] (0.64) | [-0.10, 0.06] (0.64) | [-0.24, 0.04] (0.17) | [-0.24, 0.05] (0.21) | [-0.23, 0.04] (0.16) |
| Men x Single | -0.04 | -0.02 | -0.28 | -0.21 | -0.23 |
|  | [-0.26, 0.18] (0.72) | [-0.24, 0.21] (0.88) | [-0.67, 0.10] (0.15) | [-0.62, 0.20] (0.31) | [-0.60, 0.13] (0.22) |
| ***Parenting type x Partnership status*** | | | | | |
| Lead x Academic | -0.10+ | -0.11* | -0.17+ | -0.22* | -0.18* |
|  | [-0.19, 0.00] (0.05) | [-0.20, -0.01] (0.04) | [-0.35, 0.01] (0.07) | [-0.41, -0.03] (0.02) | [-0.36, 0.00] (0.05) |
| Satellite x Academic | -0.05 | -0.07 | 0.07 | 0.01 | 0.03 |
|  | [-0.17, 0.08] (0.46) | [-0.20, 0.06] (0.28) | [-0.12, 0.26] (0.46) | [-0.19, 0.21] (0.93) | [-0.15, 0.20] (0.77) |
| Lead x Single | 0.02 | 0.05 | -0.23 | -0.15 | -0.18 |
|  | [-0.16, 0.20] (0.82) | [-0.13, 0.23] (0.60) | [-0.54, 0.09] (0.16) | [-0.49, 0.19] (0.39) | [-0.49, 0.12] (0.24) |
| Satellite x Single | -0.14 | -0.13 | -0.50 | -0.52 | -0.58* |
|  | [-0.49, 0.21] (0.42) | [-0.50, 0.23] (0.47) | [-1.14, 0.14] (0.13) | [-1.21, 0.17] (0.14) | [-1.15, -0.02] (0.04) |
| ***Gender x Parenting type x Partnership status*** | | | | | |
| Men x Lead x Academic | 0.09 | 0.08 | 0.25 | 0.28 | 0.27 |
|  | [-0.23, 0.41] (0.58) | [-0.24, 0.41] (0.61) | [-0.38, 0.88] (0.43) | [-0.37, 0.92] (0.40) | [-0.29, 0.82] (0.35) |
| Men x Satellite x Academic | 0.02 | 0.04 | -0.02 | 0.00 | 0.02 |
|  | [-0.14, 0.19] (0.77) | [-0.13, 0.21] (0.62) | [-0.29, 0.25] (0.91) | [-0.28, 0.28] (0.98) | [-0.23, 0.27] (0.85) |
| Men x Lead x Single | 0.11 | 0.12 | 0.38 | 0.31 | 0.16 |
|  | [-0.25, 0.48] (0.54) | [-0.25, 0.48] (0.54) | [-0.29, 1.06] (0.26) | [-0.37, 0.99] (0.37) | [-0.46, 0.79] (0.61) |
| Men x Satellite x Single | 0.09 | 0.09 | 0.33 | 0.43 | 0.59 |
|  | [-0.34, 0.53] (0.68) | [-0.37, 0.55] (0.71) | [-0.48, 1.15] (0.42) | [-0.44, 1.30] (0.33) | [-0.13, 1.31] (0.11) |
| Number of papers |  |  |  |  | 0.01*** |
|  |  |  |  |  | [0.01, 0.01] (0.00) |
| ***Academic age (centered)*** |  |  |  |  |  |
| 1st degree | 0.14*** | 0.14*** | 0.21*** | 0.21*** | 0.18*** |
|  | [0.14, 0.14] (0.00) | [0.13, 0.14] (0.00) | [0.21, 0.22] (0.00) | [0.21, 0.22] (0.00) | [0.17, 0.19] (0.00) |
| 2nd degree | 0.00*** | 0.00*** | -0.01*** | -0.01*** | -0.01*** |
|  | [0.00, 0.00] (0.00) | [0.00, 0.00] (0.00) | [-0.01, -0.01] (0.00) | [-0.01, -0.01] (0.00) | [-0.01, -0.01] (0.00) |
| ***Number of children*** |  |  |  |  |  |
| 1 | 0.03* | 0.03+ | 0.02 | 0.02 | 0.03 |
|  | [0.00, 0.07] (0.04) | [0.00, 0.07] (0.05) | [-0.04, 0.08] (0.52) | [-0.05, 0.08] (0.60) | [-0.02, 0.09] (0.26) |
| 3 | -0.03 | -0.03 | -0.12*** | -0.13*** | -0.14*** |
|  | [-0.07, 0.01] (0.15) | [-0.07, 0.01] (0.14) | [-0.20, -0.05] (0.00) | [-0.21, -0.06] (0.00) | [-0.20, -0.07] (0.00) |
| 4 and more | -0.09** | -0.08* | -0.24*** | -0.22*** | -0.17** |
|  | [-0.15, -0.02] (0.01) | [-0.14, -0.01] (0.02) | [-0.36, -0.12] (0.00) | [-0.34, -0.10] (0.00) | [-0.28, -0.06] (0.00) |
| Academic employment [No] | -0.16*** | -0.16*** | -0.12** | -0.11** | -0.01 |
|  | [-0.21, -0.12] (0.00) | [-0.20, -0.11] (0.00) | [-0.20, -0.04] (0.00) | [-0.18, -0.03] (0.01) | [-0.08, 0.06] (0.81) |
| Doctoral degree [No] | -0.26*** | -0.26*** | -0.31*** | -0.32*** | -0.28*** |
|  | [-0.31, -0.20] (0.00) | [-0.32, -0.20] (0.00) | [-0.41, -0.20] (0.00) | [-0.44, -0.21] (0.00) | [-0.39, -0.18] (0.00) |
| ***Discipline (ref. Social sciences)*** | | | | | |
| Arts and humanities | -0.29*** | -0.30*** | -1.71*** | -1.80*** | -1.72*** |
|  | [-0.34, -0.24] (0.00) | [-0.36, -0.25] (0.00) | [-1.82, -1.59] (0.00) | [-1.93, -1.66] (0.00) | [-1.85, -1.59] (0.00) |
| Health sciences | 0.59*** | 0.59*** | 0.76*** | 0.76*** | 0.50*** |
|  | [0.55, 0.63] (0.00) | [0.56, 0.63] (0.00) | [0.70, 0.82] (0.00) | [0.69, 0.82] (0.00) | [0.43, 0.57] (0.00) |
| Natural sciences | 0.24*** | 0.25*** | 0.37*** | 0.36*** | 0.28*** |
|  | [0.21, 0.28] (0.00) | [0.21, 0.28] (0.00) | [0.30, 0.43] (0.00) | [0.29, 0.43] (0.00) | [0.22, 0.34] (0.00) |
| Num.Obs. | 10445 | 10013 | 10445 | 10013 | 10013 |
| R2 | 0.729 | 0.704 | 0.682 | 0.653 | 0.712 |
| R2 Adj. | 0.728 | 0.703 | 0.681 | 0.652 | 0.711 |
| AIC | 22657.2 | 21822.4 | 34862.3 | 33705.1 | 31842.3 |
| BIC | 22867.5 | 22031.5 | 35072.7 | 33914.3 | 32058.7 |
| Log.Lik. | -11299.582 | -10882.206 | -17402.147 | -16823.572 | -15891.166 |
| F | 1037.098 | 877.652 | 827.928 | 696.898 | 882.488 |
| Std. Errors | Robust | Robust | Robust | Robust | Robust |

Note: Numbers in the squared brackets are confidence intervals; numbers in the parentheses are *p* values.

+ *p*<0.1, * *p*<0.05, ** *p*<0.01, *** *p*<0.001

Table S16. Comparison between respondents who finished the survey and non-respondents/non-finishers in key indicators.

|  |  | No | Yes | Total |
| --- | --- | --- | --- | --- |
| N |  | 1,386,842 | 14,629 | 1,401,471 |
| Year publishing first paper | Mean (SD) | 2009.7 (2.9) | 2009.4 (2.8) | 2009.7 (2.9) |
| Academic age | Mean (SD) | 8.6 (4.9) | 10.4 (4.1) | 8.7 (4.9) |
| Sex | Male | 760350 (54.8) | 6036 (41.3) | 766386 (54.7) |
|  | Female | 384103 (27.7) | 7037 (48.1) | 391140 (27.9) |
|  | Unknown | 242389 (17.5) | 1556 (10.6) | 243945 (17.4) |
| Discipline | Clinical Medicine | 359520 (25.9) | 3607 (24.7) | 363127 (25.9) |
|  | Engineering and Technology | 221656 (16.0) | 1032 (7.1) | 222688 (15.9) |
|  | Biomedical Research | 146968 (10.6) | 1472 (10.1) | 148440 (10.6) |
|  | Physics | 120465 (8.7) | 775 (5.3) | 121240 (8.7) |
|  | Biology | 114658 (8.3) | 1136 (7.8) | 115794 (8.3) |
|  | Chemistry | 96619 (7.0) | 551 (3.8) | 97170 (6.9) |
|  | Earth and Space | 88805 (6.4) | 851 (5.8) | 89656 (6.4) |
|  | Social Sciences | 56952 (4.1) | 1628 (11.1) | 58580 (4.2) |
|  | Professional Fields | 48684 (3.5) | 1206 (8.2) | 49890 (3.6) |
|  | Mathematics | 48791 (3.5) | 354 (2.4) | 49145 (3.5) |
|  | Health | 33341 (2.4) | 643 (4.4) | 33984 (2.4) |
|  | Psychology | 32237 (2.3) | 941 (6.4) | 33178 (2.4) |
|  | Humanities | 15571 (1.1) | 377 (2.6) | 15948 (1.1) |
|  | Arts | 2575 (0.2) | 56 (0.4) | 2631 (0.2) |
| Region | Europe & Central Asia | 532383 (38.4) | 5395 (36.9) | 537778 (38.4) |
|  | North America | 342021 (24.7) | 7535 (51.5) | 349556 (24.9) |
|  | East Asia & Pacific | 321201 (23.2) | 964 (6.6) | 322165 (23.0) |
|  | Latin America & Caribbean | 78217 (5.6) | 329 (2.2) | 78546 (5.6) |
|  | Middle East & North Africa | 48790 (3.5) | 173 (1.2) | 48963 (3.5) |
|  | South Asia | 48716 (3.5) | 100 (0.7) | 48816 (3.5) |
|  | Sub-Saharan Africa | 15514 (1.1) | 133 (0.9) | 15647 (1.1) |
| Number of papers | Mean (SD) | 25.5 (61.4) | 29.2 (52.9) | 25.6 (61.3) |
| MNCS | Mean (SD) | 37.2 (153.5) | 49.6 (155.6) | 37.3 (153.5) |

Note: For categorical variables, the unit of the values in the parentheses is percent.

**Table S17.** Logistic regression on respondents who finished the survey and non-respondents/non-finishers.

|  | Finish (Log Odds) | Finish (Odds Ratio) |
| --- | --- | --- |
| Intercept | -6.250*** | 0.002*** |
|  | [-6.323, -6.178] | [0.002, 0.002] |
| Year publishing first paper | 0.159*** | 1.173*** |
|  | [0.149, 0.170] | [1.161, 1.185] |
| Academic age | 0.165*** | 1.179*** |
|  | [0.156, 0.174] | [1.168, 1.190] |
| **Sex (ref. Male)** |  |  |
| Female | 0.784*** | 2.190*** |
|  | [0.748, 0.820] | [2.112, 2.270] |
| Unknown | 0.189*** | 1.208*** |
|  | [0.132, 0.246] | [1.141, 1.279] |
| **Discipline (ref. Clinical Medicine)** |  |  |
| Engineering and Technology | -0.212*** | 0.809*** |
|  | [-0.282, -0.141] | [0.754, 0.869] |
| Biomedical Research | -0.028 | 0.972 |
|  | [-0.089, 0.033] | [0.914, 1.034] |
| Physics | -0.127** | 0.881** |
|  | [-0.205, -0.048] | [0.814, 0.953] |
| Biology | 0.108** | 1.114** |
|  | [0.040, 0.176] | [1.041, 1.192] |
| Chemistry | -0.330*** | 0.719*** |
|  | [-0.421, -0.240] | [0.656, 0.787] |
| Earth and Space | 0.084* | 1.088* |
|  | [0.008, 0.160] | [1.008, 1.173] |
| Social Sciences | 1.123*** | 3.075*** |
|  | [1.061, 1.185] | [2.890, 3.272] |
| Professional Fields | 1.007*** | 2.736*** |
|  | [0.938, 1.075] | [2.555, 2.931] |
| Mathematics | 0.075 | 1.078 |
|  | [-0.036, 0.186] | [0.965, 1.205] |
| Health | 0.508*** | 1.662*** |
|  | [0.422, 0.594] | [1.525, 1.812] |
| Psychology | 0.790*** | 2.203*** |
|  | [0.716, 0.863] | [2.046, 2.371] |
| Humanities | 1.147*** | 3.148*** |
|  | [1.035, 1.258] | [2.816, 3.519] |
| Arts | 1.095*** | 2.990*** |
|  | [0.825, 1.366] | [2.282, 3.919] |
| **Region (ref. Europe & Central Asia)** | | |
| North America | 0.765*** | 2.149*** |
|  | [0.729, 0.801] | [2.073, 2.228] |
| East Asia & Pacific | -0.951*** | 0.387*** |
|  | [-1.020, -0.881] | [0.361, 0.414] |
| Latin America & Caribbean | -0.784*** | 0.456*** |
|  | [-0.897, -0.672] | [0.408, 0.511] |
| Middle East & North Africa | -0.775*** | 0.461*** |
|  | [-0.928, -0.623] | [0.395, 0.536] |
| South Asia | -1.186*** | 0.305*** |
|  | [-1.385, -0.987] | [0.250, 0.373] |
| Sub-Saharan Africa | -0.117 | 0.889 |
|  | [-0.291, 0.056] | [0.747, 1.058] |
| Number of papers (log) | -0.049** | 0.952** |
|  | [-0.085, -0.013] | [0.919, 0.987] |
| MNCS (log) | 0.090*** | 1.094*** |
|  | [0.067, 0.113] | [1.069, 1.120] |
| N | 1401471 | 1401471 |
| R2 | 0.894 | 0.894 |
| AIC | 147737.9 | 147737.9 |
| + p < 0.1, * p < 0.05, ** p < 0.01, *** p < 0.001 | | |

Fig S2. Comparisons between partnered academics with an academic partner (i.e., in a two-academics household) versus within in parenting related tasks (Figure 2 in the original manuscript)


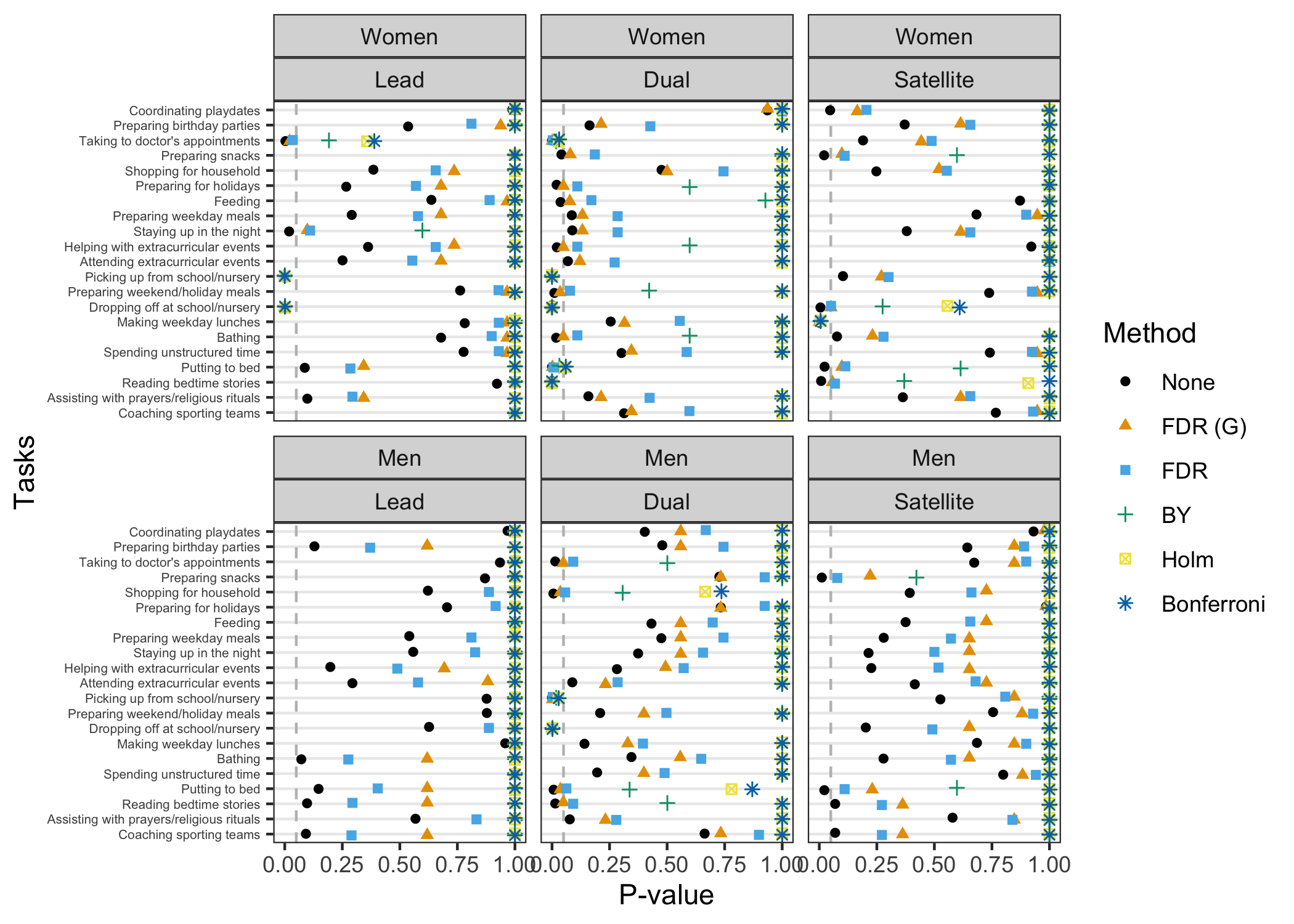


Fig S3. Gender difference in involving in parenting-related tasks within three parenting types (Figure 1C in the original manuscript)


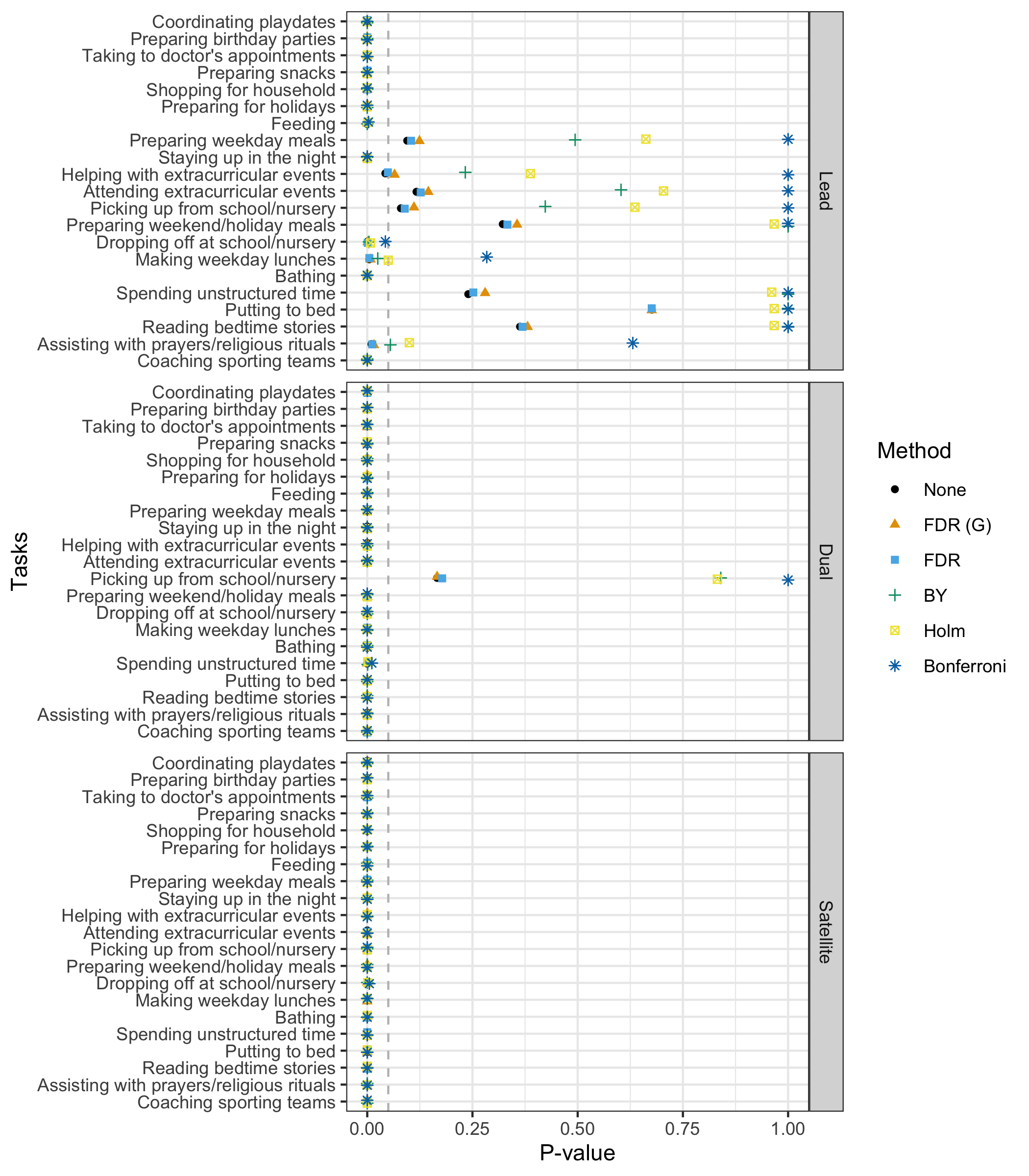


**Fig S4.** Gender difference in involving in time engagements within three parenting types (Figure 1B in the original manuscript)


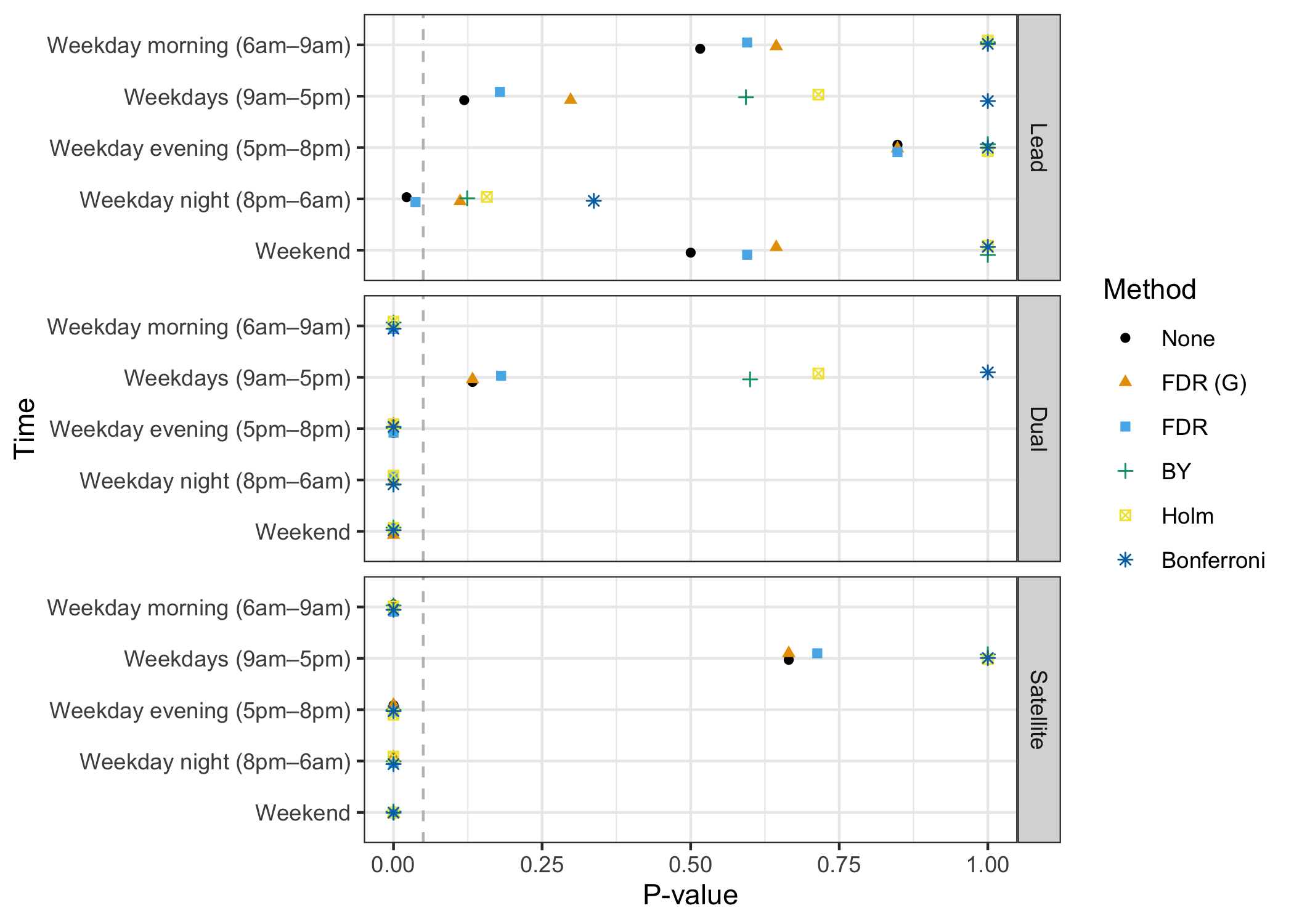


**Fig S5.** Screenshot of Stage 1 inductive coding in Nvivo of a random sample of n=1000 samples from a total of n=5976 comments


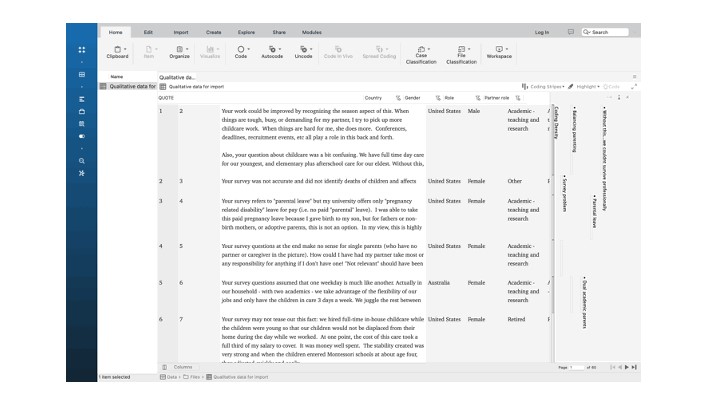


**Tabel S18.** Table showing the transitition of Stage 1 codes (Fig S5) to Stage 2 codes in preparation of coding of remaining n=4976 comments

| **Key for Stage 1 to Stage 2** | | | |
| --- | --- | --- | --- |
| **Level of comment** | **Stage 2 code** | **Stage 2 Reference** |  |
| Micro - Individual | Balancing parenting between parents (examples and opinions) | **1** |  |
| Other | Other | **2** |  |
| Micro - Individual | Comparison between men and women or childless vs parents | **3** |  |
| Meso - Organisational | Parental leave/child care policies | **4** |  |
| Micro - Individual | Career or Child affected | **5** |  |
| Micro - Individual | Choice (or lack of) | **6** |  |
| Meso - Organisational | Environmental issues eg. Academic has changed/different/flexible | **7** |  |
| Meso | Travel and effect of travel | **8** |  |

| **Number** | **Stage 1 code** | **Stage 2 code** |
| --- | --- | --- |
| 1 | Comparisons with male colleagues with parenting | 3 |
| 2 | Paid parental leave helpful | 4 |
| 3 | Prioritised children | 6 |
| 4 | Choice was right for me, despite challenges | 6 |
| 5 | Children now grown (answered retrospectively) | 2 |
| 6 | Flexible childcare policies allowed balance between work and childcare | 4 |
| 7 | Inflexible policies for later children did not allow this balance. Prioritised male careers. | 4 |
| 8 | Working parent helped child's development | 5 |
| 9 | Having children helpful for pushing work/life balance | 5 |
| 10 | Being far from extended family difficult | 7 |
| 11 | Creative childcare | 1 |
| 12 | Flexible academic careers | 7 |
| 13 | Reciprocity | 1 |
| 14 | Survey suggestions | 2 |
| 15 | Balancing childcare AND house care | 1 |
| 16 | Academia has changed | 7 |
| 17 | No paid parental care | 4 |
| 18 | Worked parttime to take over childcare | 1 |
| 19 | Maternity and parenthood had long term consequences | 5 |
| 20 | Effect of parenthood not taken into account in subsequent evaluations | 7 |
| 21 | Father's career has suffered more | 5 |
| 22 | Equal share of childcare not possible | 1 |
| 23 | Father is lead parent, mother is breadwinner | 1 |
| 24 | Support from male partner essential to success | 1 |
| 25 | Travel particularly hard | 8 |
| 26 | Costs prohibitive | 4 |
| 27 | Creative work practices | 1 |
| 28 | Guilt | 6 |
| 29 | Divorce | 1 |
| 30 | Invisible burden | 1 |
| 31 | Children after tenure | 7 |
| 32 | Left academia | 5 |
| 33 | Children are an asset | 5 |
| 34 | Breastfeeding | 4 |
| 35 | Exhaustion/Stress | 5 |
| 36 | Financial | 4 |
| 37 | Chose to restrict number of children | 6 |
| 38 | Parenting was rewarding | 6 |
| 39 | Stay at home husband | 1 |
| 40 | Wife agreed to do parenting | 6 |
| 41 | Summer breaks are challenging | 7 |
| 42 | Lack of wider support | 1 |
| 43 | Partners in the same institution | 1 |
| 44 | Wife sacrificed career | 6 |
| 45 | Career slowed | 5 |
| 46 | Husband career considered first | 1 |
| 47 | Academia and children not compatible | 7 |
| 48 | No regrets | 6 |
| 49 | Childcare is still gendered | 4 |
| 50 | Choice was right for me, despite challenges | 6 |
| 51 | Balance is a challenge | 1 |
| 52 | Career became more demanding over time | 7 |
| 53 | Childcare responsibilities change over time | 1 |
| 54 | It can be done | 6 |
| 55 | Missed parenting | 3 |
| 56 | Having children discouraged me from a research career | 5 |
| 57 | Commuting | 8 |
| 58 | Systematic discrimination | 7 |
| 59 | Never mention the child | 3 |

**Fig S6**. Screenshort of the manual Stage 2 coding process. Codes developed in Stage 1 were used to code the remaing n=4976 comments (Stage 2)

**
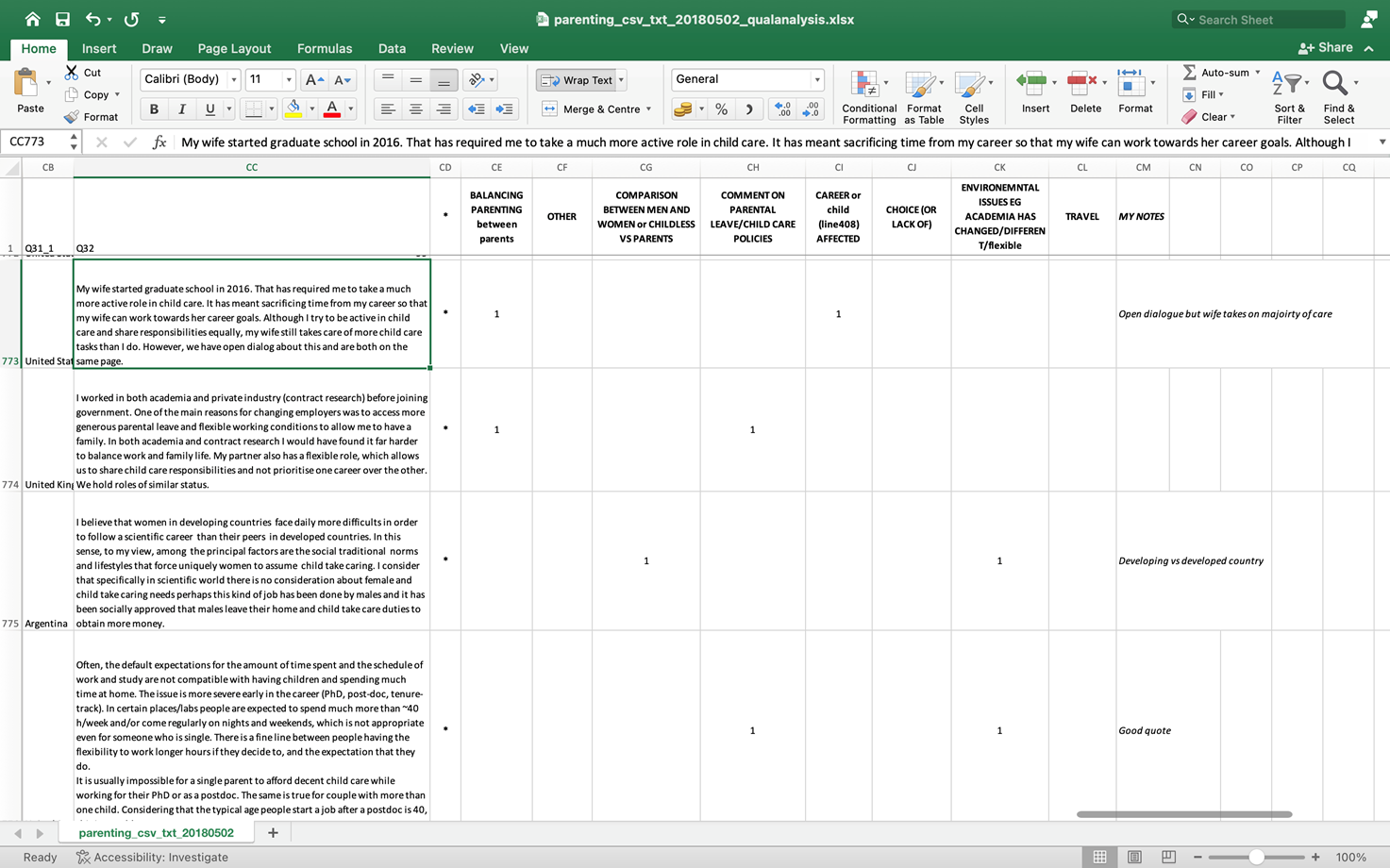
**
